# Transgenerational effects persist down the maternal line in marine sticklebacks: gene expression matches physiology in a warming ocean

**DOI:** 10.1111/eva.12370

**Published:** 2016-02-28

**Authors:** Lisa N.S. Shama, Felix C. Mark, Anneli Strobel, Ana Lokmer, Uwe John, K. Mathias Wegner

**Affiliations:** ^1^ Coastal Ecology SectionAlfred‐Wegener‐Institut Helmholtz‐Zentrum für Polar‐und MeeresforschungWadden Sea Station SyltGermany; ^2^ Integrative Ecophysiology SectionAlfred‐Wegener‐Institut Helmholtz‐Zentrum für Polar‐und MeeresforschungBremerhavenGermany; ^3^ Man Society Environment (MGU)Department of Environmental SciencesUniversity of BaselSwitzerland; ^4^ Ecological Chemistry SectionAlfred‐Wegener‐Institut Helmholtz‐Zentrum für Polar‐und MeeresforschungBremerhavenGermany

**Keywords:** acute versus developmental acclimation, climate change, epigenetics, *Gasterosteus aculeatus*, maternal effects, mitochondrial respiration, transcriptome, transgenerational plasticity

## Abstract

Transgenerational effects can buffer populations against environmental change, yet little is known about underlying mechanisms, their persistence or the influence of environmental cue timing. We investigated mitochondrial respiratory capacity (MRC) and gene expression of marine sticklebacks that experienced acute or developmental acclimation to simulated ocean warming (21°C) across three generations. Previous work showed that acute acclimation of grandmothers to 21°C led to lower (optimized) offspring MRCs. Here, developmental acclimation of mothers to 21°C led to higher, but more efficient offspring MRCs. Offspring with a 21°C × 17°C grandmother‐mother environment mismatch showed metabolic compensation: their MRCs were as low as offspring with a 17°C thermal history across generations. Transcriptional analyses showed primarily maternal but also grandmaternal environment effects: genes involved in metabolism and mitochondrial protein biosynthesis were differentially expressed when mothers developed at 21°C, whereas 21°C grandmothers influenced genes involved in hemostasis and apoptosis. Genes involved in mitochondrial respiration all showed higher expression when mothers developed at 21° and lower expression in the 21°C × 17°C group, matching the phenotypic pattern for MRCs. Our study links transcriptomics to physiology under climate change, and demonstrates that mechanisms underlying transgenerational effects persist across multiple generations with specific outcomes depending on acclimation type and environmental mismatch between generations.

## Introduction

The world′s oceans are changing at an alarming rate. Average sea surface temperatures and acidities are currently at levels that represent a major departure from the geochemical conditions that have prevailed in the oceans for up to millions of years (Hoegh‐Guldberg and Bruno 2010; IPCC 2014). The rate and scale of these changes have already resulted in consequences for marine ecosystems such as changes to species′ physiology and phenology, and to the composition and distribution of communities (Poloczanska et al. 2013). Yet, evolutionary responses could help marine species counter stressful conditions or even flourish in the face of these environmental changes (Munday et al. 2013; Reusch 2014; Sunday et al. 2014). The question is can they respond fast enough (Visser 2008; Hoffmann and Sgro 2011)? Populations can respond to rapidly changing marine environments by dispersing to suitable habitats elsewhere (e.g. latitudinal range shifts; Thomas et al. 2012), rapid evolution (Sunday et al. 2011; Lohbeck et al. 2012; Kelly et al. 2013), and/or adaptive phenotypic plasticity (reviewed in Munday et al. 2013; Reusch 2014). Phenotypic plasticity can occur both within a generation (individual responds to environment) and across generations (transgenerational; Mousseau and Fox 1998), the latter being a particularly effective mechanism as it is an inherited, fast, phenotypic response that can buffer populations against immediate impacts of climate change and provide time for genetic adaptation to catch up (Chevin et al. 2010).

Transgenerational plasticity (TGP) belongs to a broader suite of nongenetic inheritance mechanisms whereby the environment experienced by previous generations influences phenotypes in the current generation without changes to DNA sequence (Jablonka and Lamb 1995). In the case of TGP (or anticipatory parental effects *sensu* Marshall and Uller 2007), parental environment effects influence offspring reaction norms (different phenotypes expressed by the same genotype in different environments), and are manifested as a parent environment by offspring environment interaction (Mousseau and Fox 1998). Mechanisms of nongenetic inheritance include the transmission of nutrients or glandular secretions (e.g. yolk, milk), somatic factors such as hormones, cell structures (e.g. membranes, mitochondria), and epigenetic variation (e.g. DNA methylation patterns, histone modifications, or mRNAs) that can alter the physiology and phenotypes of offspring (Ho and Burggren 2010; Bonduriansky et al. 2012). There are abundant recent examples showing adaptive TGP for life history traits in marine species (e.g. Salinas and Munch 2012; papers reviewed in Salinas et al. 2013; Massamba‐N′Siala et al. 2014; Murray et al. 2014; Donelson and Munday 2015; Shama 2015; Thor and Dupont 2015; Rodríguez‐Romero et al. in press). Mechanisms on the cellular or molecular level that potentially underlie these responses are, however, often not known (but see Shama et al. 2014; Veilleux et al. 2015; DeWit et al. *in press*). Moreover, the persistence of nongenetic inheritance mechanisms across multiple generations has only rarely been investigated in marine species (Donelson et al. 2012; Shama and Wegner 2014; Donelson and Munday 2015; Thor and Dupont 2015; Rodríguez‐Romero et al. *in press*), so it is currently unclear if transgenerational effects accumulate or are reset with each generation (Shea et al. 2011; Burton and Metcalfe 2014; Herman et al. 2014).

At the cellular level, the capacity to meet increased oxygen demands and maintain aerobic scope at increased temperature will be critical in determining local population persistence in a warming ocean (Pörtner and Knust 2007). A decline in aerobic scope (the capacity for aerobic metabolism above resting metabolic rate) can affect crucial biological functions such as growth, reproduction and behaviour (Pörtner and Farrell 2008). Several recent studies have demonstrated adaptive TGP of aerobic scope in response to ocean warming and acidification (Donelson et al. 2012; Miller et al. 2012; Parker et al. 2012; Thor and Dupont 2015). However, in the majority of cases, aerobic scope was measured as a whole‐organism response, and the underlying cellular mechanism(s) was not addressed (but see Shama et al. 2014). Within the cell, mitochondria are likely to be key players due their role in bioenergetics, biosynthesis and intracellular signalling (Chandel 2014). Recent studies of temperate marine fish have demonstrated within‐generation acclimation of oxygen transport to warmer temperatures (Guderley and Johnston 1996) and maternal TGP of mitochondrial respiratory capacity (MRC) in response to simulated ocean warming (Shama et al. 2014). Given the predominant maternal inheritance of mitochondria (Brown 2008), MRCs are likely to be strongly influenced not only by maternal environment effects, but also effects from previous generations down the maternal line (e.g. maternal grandmother and beyond). Yet, to date, we have a limited understanding of how species might be able to alter their physiology in response to rapid climate change over multiple generations (Donelson et al. 2012).

Identifying the molecular processes that underlie transgenerational effects is necessary to fully assess the ability of populations to respond to rapid climate change. At the molecular level, one fundamental mechanism of phenotypic plasticity is up‐ or down‐regulation of the expression of individual genes involved in either regulative processes or directly in functional trait expression to meet the organism′s needs in a changing environment (Aubin‐Horth and Renn 2009). Transcriptomics expand upon targeted gene approaches, and have made it possible to simultaneously investigate the transcriptional response of a wide range of cellular processes, both within and across generations (e.g. epigenetics, Harms et al. 2014; Veilleux et al. 2015). For example, aerobic metabolism genes involved in thermal acclimation were shown to be up‐regulated during cold‐acclimation in temperate (Orczewska et al. 2010) and Antarctic fish species (Windisch et al. 2014), and down‐regulated during warm acclimation in *Daphnia*,* Drosophila* and yeast species (Yampolsky et al. 2014). In a tropical reef fish, gene expression patterns differed between fish exposed to elevated temperatures developmentally versus transgenerationally, with the latter showing higher expression for genes involved in cryoprotection and protein synthesis (Veilleux et al. 2015). In calanoid copepods, genes involved in RNA transcription and DNA helicase activity showed strong down‐regulation after two generations at high pCO_2_, whereas copepods exposed to intermediate pCO_2_ levels at both acute and transgenerational scales re‐attained expression levels like those seen at low pCO_2_ after reintroduction (DeWit et al. *in press*). These recent studies demonstrate that gene expression profiles can differ across generations depending on the strength and duration (number of generations) of the changing climate cue, and may lead to increased tolerance or susceptibility of populations to rapidly changing environmental conditions.

Missing from the current TGP literature are investigations into the importance of *when* an organism is exposed to environmental cues signalling a changing climate, and the consequences for future generations. Environmental influences on transgenerational effects can be driven by cues experienced by the parental generation as adults during reproductive conditioning (acute acclimation) or by the environment experienced by parents during early life, from fertilization to the end of juvenile growth (developmental acclimation; Burton and Metcalfe 2014). Examples of acute acclimation effects, particularly maternal environment effects, are pervasive in the literature (Mousseau and Fox 1998; Marshall and Uller 2007; Räsänen and Kruuk 2007), and these acute effects during critical reproductive periods can influence offspring phenotypes via various nongenetic pathways (see above). Perhaps even more striking are studies showing that environmental effects experienced around conception or early life of parents can produce particularly strong transgenerational effects due to the fact that early embryonic cells are more sensitive to environmental conditions, and that epigenetic alterations at this point in time affect a higher proportion of cells, including germline cells (Shea et al. 2011; Burton and Metcalfe 2014; Herman et al. 2014). Two recent studies have shown that transgenerational effects differ depending on acute versus developmental acclimation of parents (Shama and Wegner 2014; Donelson and Munday 2015), and that the critical window for environmental cues can vary between species. In both studies, whole‐organism responses were measured (growth and offspring sex ratio, respectively), so we still lack information about whether the cellular and molecular mechanisms involved in the two types of acclimation differ, and if these mechanisms are stable across multiple generations.

Here, we investigated cellular and molecular mechanisms potentially underlying transgenerational effects to ocean warming, and if these differ depending on the type of acclimation (acute versus developmental) of grandparental and parental generations of three‐spined stickleback (*Gasterosteus aculeatus* Linnaeus 1758). The studied population inhabits an area of the North Sea with a mean annual sea surface temperature of 17°C during summer months (Ramler et al. 2014; Schade et al. 2014). We simulated a warming ocean by exposing fish to a temperature increase of 4°C, which is predicted by various model scenarios for global change (Sheppard 2004; IPCC 2014). Since our temperature manipulations were within the natural variability range experienced by the population (Shama 2015), they represent a relevant magnitude of environmental change in a future ocean scenario (Donelson et al., unpublished manuscript). Our previous work showed that acute acclimation of wild‐caught sticklebacks (F0) to elevated temperature during reproductive conditioning led to strong maternal TGP benefits on F1 offspring growth, with a matching pattern for MRC (Shama et al. 2014). Developmental acclimation of F1 mothers to elevated temperature, however, led to negative maternal effects on F2 offspring growth, yet positive maternal grandmother effects remained (Shama and Wegner 2014). Since the maternal environment effects in the two generations influenced offspring phenotypes in opposing directions, we suggested that the mechanism of information transfer might have differed depending on the type of acclimation (Shama and Wegner 2014).

In the current study, we used F2 sticklebacks from the experimental setup of Shama and Wegner (2014), and followed the inheritance of mitochondria, looking specifically at the interaction between maternal grandmother and maternal (dam) environments on F2 offspring MRC and gene expression (transcriptome) profiles. By taking such an approach, we can determine (i) whether TGP of mitochondrial respiration also occurs when mothers experience developmental acclimation, (ii) which genes and cellular processes are involved in thermal acclimation and if these are differentially expressed across generations depending on the type of acclimation, and (iii) if expression profiles of mitochondria encoded genes correspond to our phenotypic measures of mitochondrial respiration. Our study aims to link transcriptomic responses to physiology in a changing marine climate to shed light on the mechanisms involved in transgenerational effects and their persistence over multiple generations. By doing so, we can identify critical developmental phases when organisms are most susceptible to climate change. Moreover, by applying a factorial experimental design to transcriptomic analyses, we can disentangle the effects of persistent environmental stress over multiple generations from environmental mismatch between generations on key genes and cellular processes involved in thermal acclimation. Such an approach has not been used in climate change studies to date, but is a promising avenue for future studies of transgenerational effects in response to not only directional changes in climate over generations, but also to the predicted increase in climate variability (IPCC 2014) that may result in a higher frequency of mismatches between generations.

## Materials and methods

### Experimental design

Grandparent sticklebacks originated from an oceanic population caught in the Sylt‐Rømø Bight, Germany (55°05′N, 8°41E) in February 2012. Wild adult fish (F0) were brought to the laboratory and held at 17°C or 21°C for 2 months during reproductive conditioning (acute acclimation). In May 2012, we produced pure crosses within and reciprocal crosses between acclimation temperatures, and reared F1 offspring at both temperatures (see Shama et al. 2014 for details). After 60 days, F1 families were pooled within each sire‐dam‐offspring temperature combination group (8 groups in total; G1–G8; Table S1). Each group was divided among replicate 25* *L aquaria, maintained at their offspring temperature (4 groups at 17°C; 4 groups at 21°C), and reared to adulthood. Offspring growth at 30 and 60 days posthatch, and mitochondrial respiratory capacity of adults was measured on F1 fish (Shama et al. 2014).

In March 2013, we made F2 crosses among the G1–G8 groups to produce F2 families in 15 temperature combinations (see Shama and Wegner 2014 for details). In each combination, F1 sires and dams experienced developmental acclimation at either 17°C or 21°C. We produced pure crosses (parent and grandparent temperatures the same) and mixed reciprocal crosses (parent and grandparent temperatures differed), and reared offspring at both temperatures (Table S1). F2 families were reared individually and offspring growth was measured at 30, 60 and 90 days posthatch (Shama and Wegner 2014). In October 2013, F2 families were pooled according to their G group × G group cross combinations (e.g. 3 families in G1 × G1; Table S1) to a maximum density of 30 fish per 25 L aquarium, maintained at their offspring temperature, and reared to adulthood. Note: G × G groups with less than 25 fish in total were divided amongst 2 L aquaria with a maximum density of 2–3 fish per aquarium so that all fish experienced a density of approximately 1 fish per L water. In June 2014, we produced eight groups of adult F2 fish within each maternal granddam (MGD)‐dam‐offspring temperature combination (R1–R8; Table S1), as we were interested in the path of mitochondrial inheritance down the maternal line in the current study. Approximately 20 fish per R group (except groups R7 and R8: *n* = 10 and *n* = 5, respectively) were randomly selected from all corresponding GxG groups (Table S1), transferred to 25 L aquaria and maintained at their offspring rearing temperature until the mitochondrial respiration assays were performed (August 2014). Although the lack of replicate aquaria for R groups is a potential concern (see Cornwall and Hurd 2015), we do not think it contributed to any pre‐existing bias in temperature effects, as each R group consisted of multiple G × G groups, and the F2 adults used were a random selection representing accumulated variation among replicates for each treatment across all previous stages of the experiment. Throughout the study, fish younger than 90 days old were fed daily with Artemia sp. larvae *ad libitum*, after which they were fed daily with chironomid larvae *ad libitum*.

### Mitochondrial respirometry and data analyses

We used adult F2 fish to ensure that sufficient amounts of heart tissue were available for the respiration assays. Respiration assays were performed on 18–21 August 2014. For each assay, six adult fish were randomly selected from within each of groups R1–R6, and assays were replicated three times. At the time of the mitochondrial assays, only seven fish remained in group R7 (21°C MGD × 21°C dam reared at 17°C) and one fish in R8 (21°C MGD × 21°C dam reared at 21°C), hence, only one assay replicate was performed on R7 and none for R8. Assays were performed as in Shama et al. 2014. Briefly, fish were sacrificed in an excess of MS‐222, standard length was measured (±1 mm), and hearts were dissected out under a binocular microscope. At the same time, pectoral fin muscle (oxidative red muscle) was dissected from both pectoral fins, flash frozen in liquid nitrogen and transferred to −80°C for subsequent transcriptomic analyses. Heart tissue from the six fish per group was first pooled, then divided into halves, and assayed at both 17°C and 21°C. In total, 38 assays were performed.

For each assay, heart fibres were held in modified assay medium (MiRO5) prior to analysis. Mitochondrial respiration was measured in 2 mL assay medium plus 300 U mL^−1^ catalase (MiRO6) in glass chambers of two Oroboros Oxygraph‐2k™ respirometers (Oroboros Instruments, Innsbruck Austria). Heart fibre respiration was converted to pmol O_2_ s^−1^mg_fresh weight_^−1^. Resting respiration (state II), maximum respiration (state III), maximum capacity of the phosphorylation system (max. OXPHOS), LEAK respiration (state IV, waste of mitochondrial substrates), maximum capacity of the electron transport system (ETS), and nonmitochondrial respiration (ROX) were measured as in Shama et al. 2014 (see also Strobel et al. 2013 for further details). General linear models with stepwise selection (minimum adequate models) were fit for OXPHOS, ETS and LEAK using MGD temperature, dam temperature, offspring temperature, and assay temperature as fixed effects. As we did not have a full factorial design due to the missing R8 group, the 4‐way interaction term and 3‐way MGD × dam × offspring term were excluded from the models. All analyses were run in the R statistical environment (R Development Core Team, 2015).

### RNA library construction

Whole RNA was extracted from approximately 30 mg pectoral fin muscle tissue from six fish from each of groups R1–R7 (*n* = 42 fish in total) using a phenol/guanidine‐based lysis reagent (QIAzol) and following the standard extraction protocol in QIAGEN RNeasy extraction kits (QIAGEN, Hilden, Germany). Total RNA concentrations were measured using a NanoDrop ND‐1000 spectrophotometer (Peqlab (VWR), Erlangen, Germany). The quality and concentration of the extracted RNA were further checked with an Agilent 2100 Bioanalyzer using the Agilent RNA 6000 Nano Kit (Agilent Technologies, Waldbronn, Germany). The dual‐indexed libraries were prepared from 1 μg RNA per sample with the TruSeq Stranded mRNA HT Sample Prep Kit (Illumina, San Diego, CA, USA). The quality and concentration of the resulting libraries was checked with an Agilent 2100 Bioanalyzer using the Agilent DNA 7500 Nano Kit (Agilent Technologies, Waldbronn, Germany). All of the above kits were used according to the manufacturers’ protocols. Data on DNA fragment length and concentration were then used to calculate the molarity of individual libraries. These were subsequently pooled equimolarly (10 nm) and sequenced (75 bp single‐end) on an Illumina NextSeq500 sequencer at the Alfred Wegener Institute, Bremerhaven, Germany. Proprietary Illumina BCL files were converted to fastq files and de‐multiplexed using bcl2fastq (v2.17, Illumina, San Diego, CA, USA) using default settings. Trimmomatic (Bolger et al. 2014) was used to remove short (<36 bp) and low quality reads (sliding window option) as well as adapters if still present. The quality of the trimmed data was analysed and confirmed with FastQC (Andrews 2010). The fastq files containing reads from the same sample but different lanes were combined into a single file before proceeding to the mapping step.

### Transcriptome assembly and gene expression analyses

After an additional trimming step removing biased leading and trailing nucleotides and short sequences (<50 bp), reads were mapped against the ensembl BROADS S1 stickleback genome assembly v82 using the RNAseq workflow of CLC Genomics workbench v8.5.1 (CLC bio, Arhus, DK). We retained only uniquely mapped reads for downstream analysis using the DESeq2 package (Love et al. 2014) in the R statistical environment (R Development Core Team 2015). We fitted the same model used for the respiration analyses calculating log2FoldChanges (LFC) per gene as a function of offspring (O), dam (D) and maternal granddam (MGD) thermal environments (21°C vs 17°C), plus their respective two‐way interactions (LFC ~O + D + MGD + O:D + O:MGD + D:MGD). LFC was calculated for each of the main and interaction effects, and statistical significance was determined based on false discovery rate (FDR) adjusted *P*‐values <0.05 (Benjamini and Hochberg^1995^) and a minimum up‐ or downward LFC of 1.

To identify biological processes significantly regulated within each contrast, we performed an enrichment analysis based on biological processes of the GO ontology using the topGO package (Alexa and Rahnenfuhrer 2010) against a background of random genes showing similar expression profiles, and identified genes responsible for enrichment of terms differentiating the different treatment groups. For broadly enriched terms like ‘metabolic process’, we focussed on genes involved in mitochondrial and energy metabolism in general. We then combined all genes with significant LFC changes in each of the contrasts into one dataset to visualize gene expression changes over all treatments. LFC values were clustered using complete linkage of Euclidean pairwise distances over treatments as well as genes, from which we identified functional clusters of genes based on the shared similarity of expression profiles. Our ultimate aim was to investigate how thermal acclimation history translates into mitochondrial respiratory capacity (MRC) on the molecular transcriptional level. Therefore, we looked specifically at transcript levels of mitochondria encoded genes. Since mitochondria encoded genes have much higher transcription levels than nuclear genes, the DESeq2 approach of fitting log2‐fold changes has its limitations, and significant regulation of mitochondrial genes might be missed against the background of nuclear genes. Therefore, we combined a differential expression approach conducted solely for mitochondria encoded genes with an analysis of normalized log transformed transcript counts for each treatment group reflecting the analyses for mitochondrial respiration parameters.

## Results

### Mitochondrial respiration

**Figure 1:**
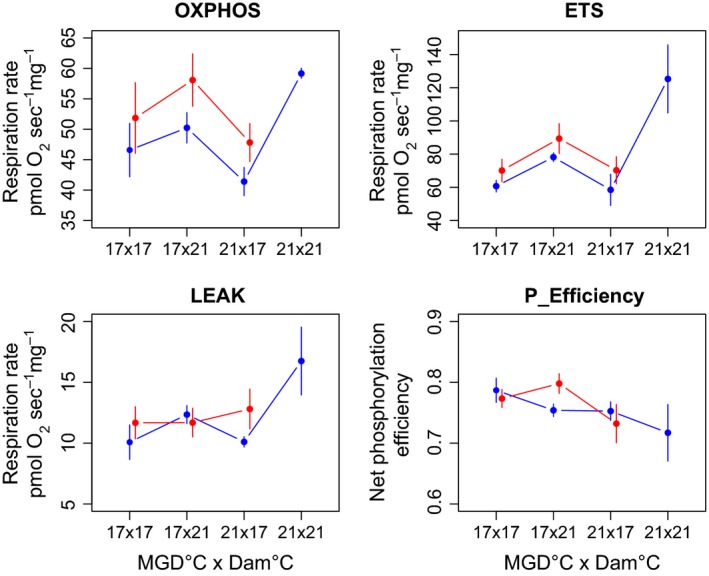
Offspring respiration rates (pmol O_2_ s^−1^ mg^−1^) measured as OXPHOS, ETS and LEAK, and net phosphorylation efficiency (P_Efficiency) for each maternal granddam (MGD) × dam temperature combination. Blue lines show offspring reared at 17°C and red lines show offspring reared at 21°C. Points depict means ± SE for each temperature combination group.

Maternal developmental temperature (dam °C) had a significant effect on all three offspring mitochondrial respiration parameters (Table 1), in that overall rates were higher when mothers developed at 21°C than at 17°C (Fig. 1A–C). Grandmother thermal environment (MGD °C) as a main effect did not have a significant influence on respiration parameters (Table 1). For example, respiration rates of offspring with a 21°C MGD × 17°C dam thermal history did not differ significantly from those with a 17 × 17 MGD × dam °C acclimation background (Fig. 1A–C). Yet, for ETS and LEAK (a measure of phosphorylation inefficiency), we detected a significant MGD x dam interaction (Table 1). For both parameters, respiration rates were highest when both MGDs and dams had 21°C acclimation backgrounds, whereas ETS and LEAK showed different patterns of response in the mismatched MGD × dam °C treatments (Fig. 1B,C). A post‐hoc analysis of net phosphorylation efficiency (calculated as 1‐(LEAK/OXPHOS)) using the same model terms as above showed an interaction between dam and offspring temperatures (*F*_1,31_ = 4.611; *P* = 0.040), with higher net efficiencies when both mothers and offspring were acclimated to 21°C (Fig. 1D). Overall, net phosphorylation efficiency was higher when assayed at 17°C than 21°C, and when MGDs were acclimated to 17°C (*F*_1,31_ = 7.96; *P* = 0.008, *F*_1,31_ = 7.94; *P* = 0.008, respectively). Offspring rearing temperature as a main effect did not have a significant influence on OXPHOS, ETS or LEAK (Table 1). ETS and LEAK were higher when assayed at 21°C than at 17°C as predicted by *Q*_10_ relationships (an increase in biological reactions with temperature), but there were no significant interactions between assay temperature and other model terms (Table 1). Since the overall patterns were qualitatively similar (e.g. respiration rates were higher for offspring of 21°C mothers at both assay temperatures), we combined assay temperatures in Fig. 1 to better highlight the main influence of thermal acclimation history.

**Table 1:** Minimum adequate models for *Gasterosteus aculeatus* mitochondrial respiration parameters (a) OXPHOS, (b) ETS and (c) LEAK depicting the influence of maternal granddam (MGD), dam, offspring and assay temperature (°C). Significant terms are highlighted in bold

	d.f.	MS	*F*	*P*
(a) OXPHOS				
MGD °C	1	221.18	2.613	0.115
Dam °C	1	386.48	4.567	**0.040**
Offspring °C	1	292.13	3.452	0.072
MGD x Dam °C	1	212.97	2.516	0.122
Residuals	33	84.64		
(b) ETS				
MGD °C	1	20.1	0.083	0.776
Dam °C	1	5965.1	24.550	**<0.001**
Offspring °C	1	509.0	2.095	0.159
Assay °C	1	1796.3	7.393	**0.011**
MGD × Dam °C	1	2959.8	12.182	**0.002**
MGD × assay °C	1	0.6	0.003	0.960
Dam × assay °C	1	0.0	0.001	0.991
Offspring × assay °C	1	450.8	1.855	0.184
MGD × dam × assay °C	1	766.5	3.155	0.087
Residuals	28	243.0		
(c) LEAK				
MGD °C	1	5.165	0.759	0.391
Dam °C	1	32.834	4.822	**0.036**
Offspring °C	1	7.074	1.039	0.316
Assay °C	1	54.535	8.010	**0.008**
MGD × dam °C	1	29.097	4.273	**0.047**
Dam × offspring °C	1	15.730	2.310	0.139
Offspring × assay °C	1	14.145	2.077	0.160
Residuals	30	6.809		

### Transcriptome profiles

**Figure 2:**
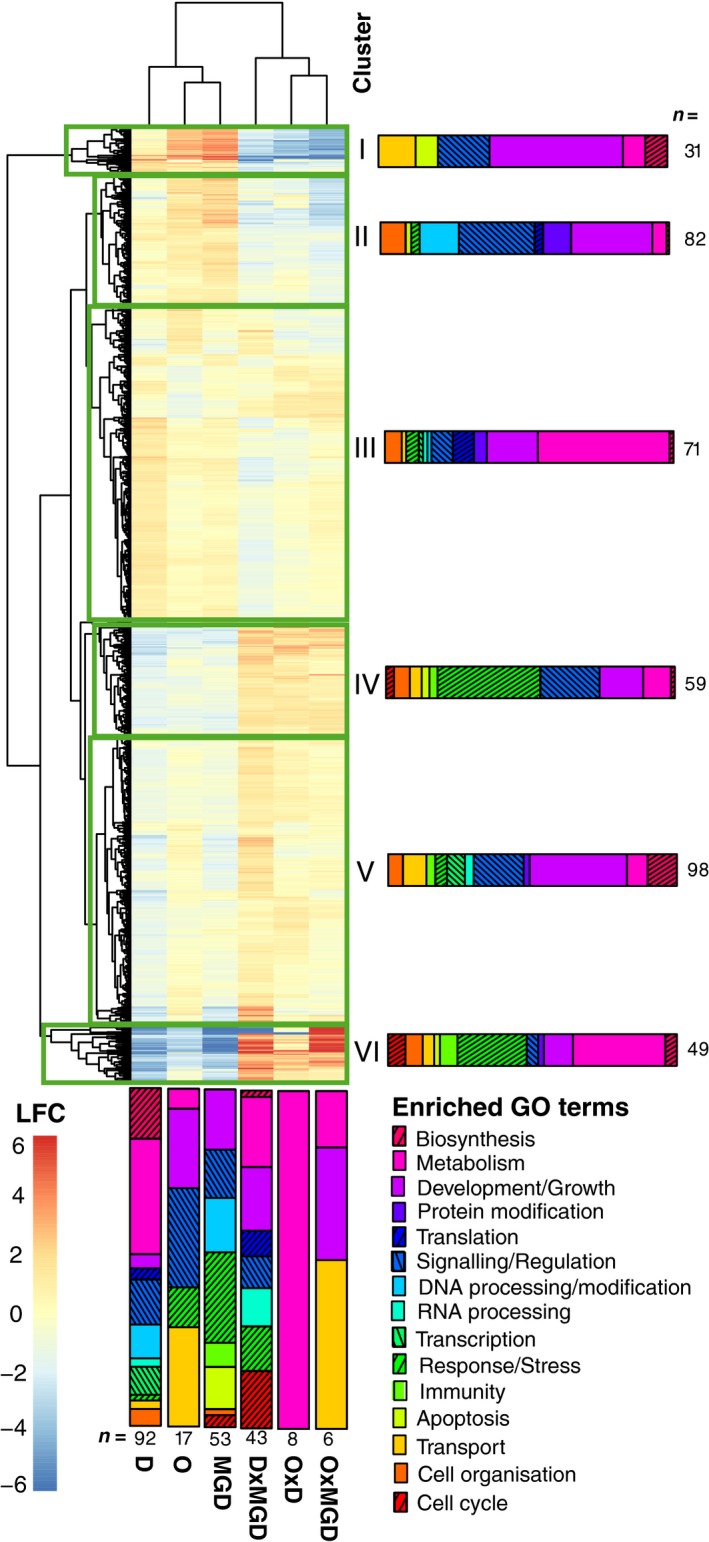
Influence of offspring rearing environment (O), and thermal acclimation history of mothers (D) and maternal grandmothers (MGD) on differential gene expression of F2 offspring. Differential expression is expressed as log2 fold changes (LFC) of 21°C versus 17°C across all main effects (O, D, MGD) and their two‐way interactions (OxD, OxMGD, DxMGD). All genes significantly regulated at a false discovery rate adjusted P‐value < 0.05 in one of these contrasts are shown. For clarity, LFC values were capped at 6 and ‐6. Clusters representing genes with similar expression profiles across treatments are marked by boxes. Classification of GO biological process terms are given for each experimental group and gene cluster, with the number of enriched GO terms given below/next to the bars.

From the 41 successfully sequenced libraries we obtained a total of 242 487 801 reads, of which 148 949 266 reads could be uniquely mapped to gene models, representing an average coverage of 3 632 909 (±919 554) mapped reads per library (Table S2). Immediate thermal (rearing) environment of the offspring (O), thermal acclimation history of mothers (D) and maternal grandmothers (MGD), and their two‐way interactions had differential effects on gene expression patterns. In total we found 2101 genes that were differentially expressed with log2 fold changes greater than one (Fig. 2).

**Figure 3:**
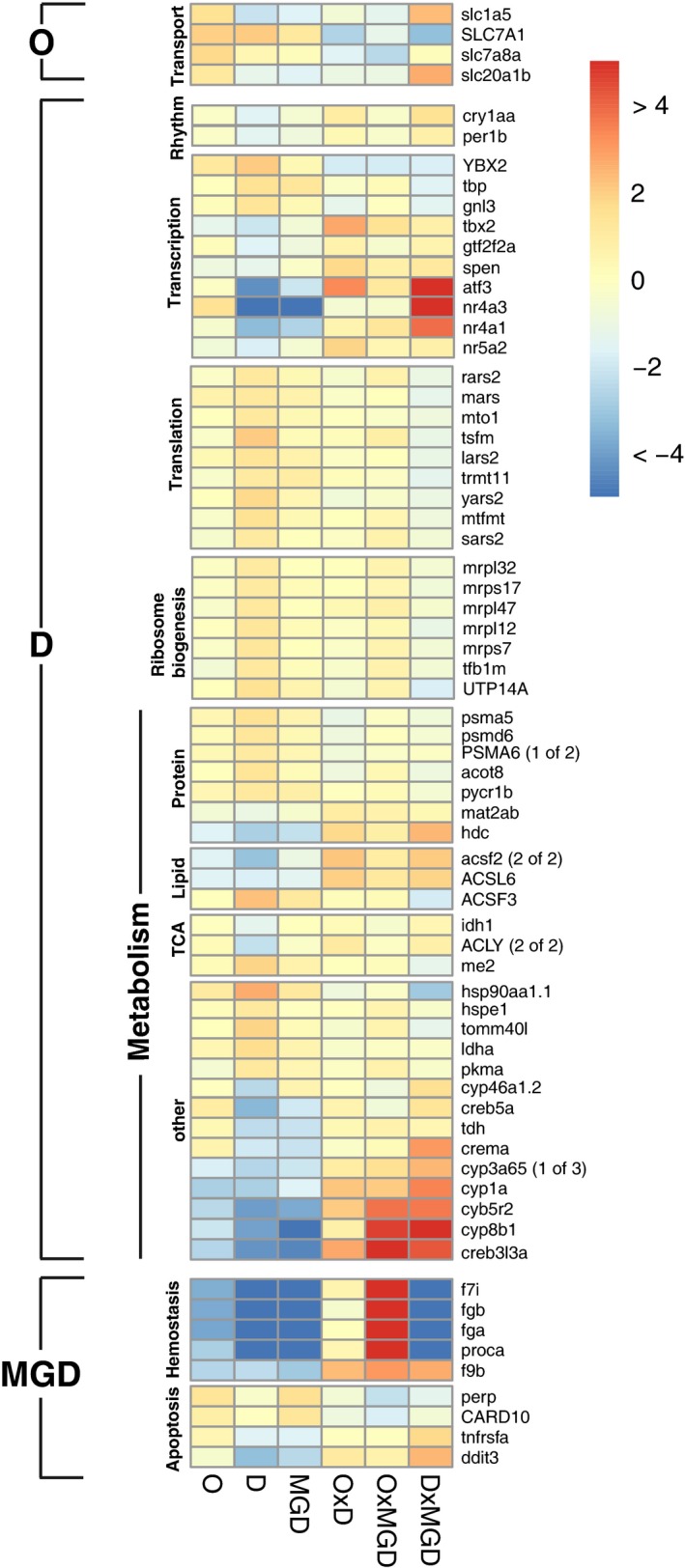
Differential expression (LFC 21°C vs 17°C) across treatments for genes identified by exclusively enriched GO‐terms within each treatment factor (O = offspring environment, D = maternal environment, MGD = grandmaternal environment). LFC values are capped at ± 4.

Maternal environment had the strongest influence on gene expression of offspring, with 1276 differentially transcribed genes from 92 enriched processes. The majority of these genes were associated with metabolic processes (34%) and biosynthesis (15%). A large number of these processes were, however, tightly interwoven with RNA processing and ribosome assembly in the mitochondria, making maternal environment the only main factor significantly enriched for transcription and translation in the energy producing organelles of the offspring (Figs 2, 3, Table S3). Genes involved in mitochondrial ribosomal biogenesis (e.g. up‐regulation of several mitochondrial ribosomal proteins *mrpl22*,* mrpl12*,* mrpl47*,* UTP14A* and ribosomal transcription factors *tfb1 m*,* gnl3*), as well as several processing genes of mitochondrial tRNAs were consistently up‐regulated when mothers developed at higher temperatures, and did not show strong interactions with the other treatments. Nuclear transcription factors, on the other hand, showed a much more variable picture. While some general transcription factors like *YBX2*,* tbp* and *gnl3* were up‐regulated, other transcription factors (*atl3*), transcription repressors (*spen*), and nuclear receptor genes (e.g. *nr4a3*,* nr4a1*) showed transgenerational down‐regulation, with partly strong interactions when maternal and grandmaternal environments did not match (Fig. 3). While some gene transcripts from maternally enriched protein and lipid metabolic processes were found in higher abundances, an equally high proportion were down‐regulated (15 out of 27) when mothers developed at 21°C. Among up‐regulated genes were also chaperones involved in protein folding (e.g. *HSP90*,* HSP10*) and genes involved in protein catabolism (e.g. proteasome genes *psma5*,* psmd6* and *PSMA6*), indicating that higher protein synthesis also led to overall higher protein turnover. Several cytochrome genes were strongly down‐regulated in almost all main factor treatments (O, D and MGD), whereas environmental mismatches within the thermal history (i.e. interactions) led to a strong induction of these genes (Figs 2, 3). Notably, maternal environment also led to down‐regulation in combination with a moderate up‐regulation in D × MGD environments of genes controlling circadian rhythm (e.g. *perb1b*,* cryaa*, Fig. 3).

Maternal granddam environment significantly influenced the expression patterns of 677 genes that were enriched in 53 GO terms belonging to stimulus response (26%, e.g. nuclear receptors *nr4a1*,* nr4a3*,* nrlh5*) and hemostasis, all of which were strongly down‐regulated when grandmothers experienced acclimation to elevated temperature (Figs 2, 3). Another process that was exclusively enriched due to MGD environment was apoptosis. Characteristic genes (*perp*,* CARD10*,* tnfrsfa*,* ddit3*) showed both up‐ and down‐regulation patterns interacting between maternal and MGD environments (Fig. 3). Offspring environment led to differential expression in 369 genes that were significantly enriched in 17 GO terms mainly associated to G‐protein signalling (e.g. *LGR4*,* gpr161*) and transport (e.g. *slc1a5*,* SLC7A1*,* scl20a1b*; Figs 2, 3; Tables S3 and S4).

Mismatches in thermal acclimation history between generations led to differential expression of fewer genes as compared to that of main treatment effects. Specifically, the interaction between offspring and maternal environment (O x D) showed differential expression of only 49 genes, while 152 genes differed between offspring and grandmother environments (O × MGD). The interaction between maternal and grandmaternal environments (D × MGD), however, was particularly strong, with 514 differentially regulated genes underlining the strong transgenerational effects governing gene expression patterns. This pattern was reflected in the enriched GO terms, showing only few transport and metabolism‐related terms in offspring interactions, while D × MGD interactions shared the majority of terms with the maternal and grandmaternal enrichment patterns (Fig. 2, Table S3).

We could form six clusters of genes based on similarity of expression patterns between the treatment groups, partly reflecting not only expression differences between the treatments, but also coherent functional groups (Fig. 2). Cluster I, for example, contained a large proportion of genes involved in muscle development that showed strong transgenerational induction, but also strong down‐regulation in D × MGD interaction environments. Cluster III contained most of the genes involved in nucleic acid metabolism enriched from the maternal environment, but was also enriched for transmembrane transport, representing up‐regulated genes depending on the offspring environment. Cluster IV contained genes enriched for several processes involved in perception and regulation of responses to various stimuli. Cluster V was strongly enriched in genes regulating various metabolic processes that showed moderate induction in interacting environments, whereas genes belonging to cluster VI showed strongly divergent expression patterns between main and interaction effects. Genes belonging to Cluster VI were involved in lipid metabolism and wounding responses/hemostasis that were also enriched by strong down‐regulation in 21°C maternal environments (Fig. 2, Table S3).

### Genes underlying mitochondrial respiration

**Figure 4:**
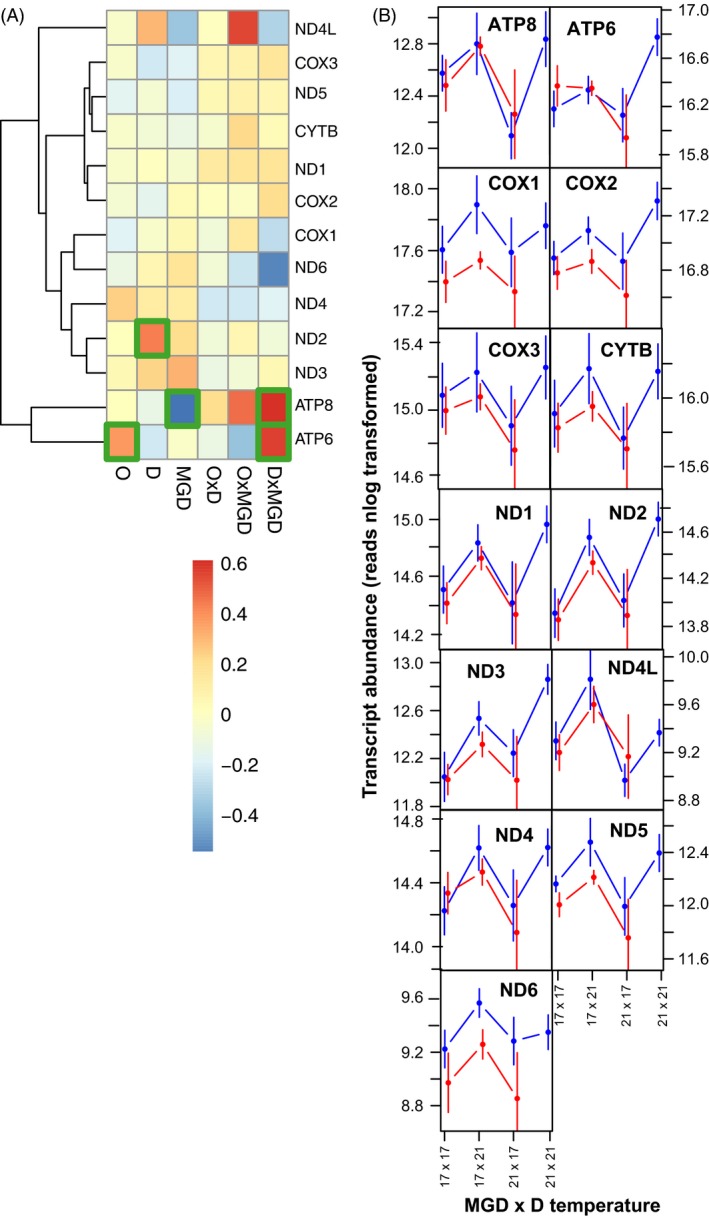
(A) Differential expression (LFC 21°C vs 17°C) of mitochondrial genes across treatments (O = offspring environment, D = maternal environment, MGD = grandmaternal environment). Genes showing significantly different expression patterns between thermal environments are highlighted with green boxes. (B) Transcript levels of mitochondrial genes as a function of maternal (M) and grandmaternal (MGD) envrionments. Blue lines show offspring reared at 17°C and red lines show offspring reared at 21°C.

The 13 mitochondria encoded genes showed much higher transcript levels than nuclear genes (e.g. as high as 461′249 reads mapped to *COX1*, Table S2). Such high expression levels make detection of significant LFC changes more difficult. Nevertheless, we found significant up‐regulation of ATP6, ATP8 and ND2, but only a single case of significant down‐regulation of ATP6 depending on MGD thermal environment. Notably, a mismatch between maternal and grandmaternal environments led to consistent up‐regulation of both ATP‐synthetase genes (Fig. 4A). When looking at transcript levels directly, it becomes clear that all mitochondria encoded genes were up‐regulated when mothers developed at 21°C, linking the up‐regulation of mitochondrial translational machinery encoded in the nucleus to actual protein production in the mitochondria (Fig. 4B).

## Discussion

The most salient finding of our study is that maternal environment had the strongest influence on offspring phenotypes. Maternal transgenerational effects spanned multiple levels of biological complexity, influencing offspring gene expression, mitochondrial respiration, and growth. Still, enduring influences of grandmaternal environment indicate that transgenerational effects can persist for multiple generations. Differential outcomes of acute versus developmental acclimation suggest that pathways underlying the transfer of information between generations may differ depending on *when* climate change cues are perceived. Moreover, environmental mismatch between generations led to very different effects on offspring phenotypes depending on whether grandmothers or mothers experienced thermal stress.

### Acclimation of mitochondrial respiratory capacity

Mitochondria are of particular interest in questions of thermal acclimation, primarily because of their role in energy metabolism. For instance, cold acclimation has been shown to enhance muscle oxidative capacity for several temperate fish species (including three‐spined stickleback), whereas warm acclimation often leads to reduced aerobic capacities (Guderley and St‐Pierre 2002). Here, we found that offspring MRCs were strongly influenced by the thermal acclimation history of their mothers and maternal grandmothers. Maternal environment effects strongly influenced mitochondrial capacity such that offspring of mothers that developed at elevated temperature had higher mitochondrial respiration rates than offspring of mothers that developed at ambient temperature. Furthermore, higher net phosphorylation efficiencies and lower associated waste of mitochondrial substrates/membrane potential (LEAK) suggest that offspring MRCs were higher but more efficient at 21°C when mothers developed at 21°C. Interestingly, offspring with 21°C × 17°C grandmother‐mother environment mismatch showed some metabolic down regulation or even compensation with respect to heart mitochondrial capacities, in that their maximum capacities were as low as fish with a 17°C thermal history across three generations. We previously associated lower OXPHOS capacities with optimized metabolic rates that can generate a higher scope for growth in temperate sticklebacks acclimated to warmer temperatures across generations (Shama et al. 2014), and this has also been observed for tropical reef fish (Donelson et al. 2012). In the current study, higher MRCs for offspring of 21°C mothers matches their pattern of smaller body sizes found in the F2 growth experiment. Likewise, optimized (lower) mitochondrial metabolism of F2 offspring in the 21°C × 17°C group is consistent with the lingering positive 21°C MGD effects previously shown for F2 offspring size (Shama and Wegner 2014). Still, here we found that offspring reared at 17°C with a cumulative 21°C thermal acclimation history (MGD and dam) had the highest respiration rates and lowest efficiencies of all groups. Unfortunately, we cannot know if offspring with a persistent 21°C thermal history would perform even worse or show metabolic compensation or higher efficiencies that would match their (relatively) larger sizes found in the F2 growth experiment (Shama and Wegner 2014). Overall, maternal environment effects appear to have the strongest influence on offspring MRC. Nevertheless, lingering MGD environment influences suggest that transgenerational effects can persist across multiple generations.

Whether transgenerational effects differ depending on acute versus developmental acclimation of previous generations has rarely been investigated (but see Shama and Wegner 2014; Donelson and Munday 2015). In sticklebacks, acute acclimation during reproductive conditioning resulted in lower (optimized) MRCs and larger sizes at warmer temperatures, suggesting that less energy was spent on maintenance metabolism and more on growth (Shama et al. 2014). In the F2 generation, smaller offspring of mothers that experienced developmental acclimation to elevated temperature spent more energy on mitochondrial metabolism, but it was more efficient. Indeed, a reduction in body size is a common finding in climate change studies (Daufresne et al. 2009), and evidence is accumulating to support the argument that ′bigger is not always better in harsh environments′ (Kaplan 1992). Whether more efficient physiological processes underlie this is not well‐studied, but the two in combination would allow for energetic savings that could be used to cope with a warming environment. At the physiological level, we previously suggested that ′primed′ mitochondria were transmitted from mothers to offspring (*sensu* structural inheritance in Ho and Burggren 2010) when mothers experienced acute acclimation to warmer temperature (Shama et al. 2014). While these ′primed′ mitochondria may or may not be inherited over multiple generations, it does not appear that they are produced when mothers experience developmental acclimation. Rather, developmental acclimation seems to lead to more general and encompassing changes to cellular metabolism (Guderley and St‐Pierre 2002). Moreover, developmental acclimation in the current generation may override the effects of acute acclimation in previous generations, as evidenced by the different directions of MRC response depending on grandmother‐mother environment combinations.

One potential pathway for the more general changes to cellular metabolism suggested here is differential resource allocation by mothers, for instance, egg size plasticity (Mousseau and Fox 1998; Bownds et al. 2010; Liefting et al. 2010). Our previous studies showed that egg size plasticity occurred when mothers experienced developmental but not acute acclimation. Mothers that developed at warmer temperature produced more, but smaller eggs, and initial egg size likely had a strong influence on offspring growth (Shama et al. 2014; Shama and Wegner 2014; Shama 2015; see also Hollowed et al. 2013; Dahlke et al. 2016). Also, although we did not investigate it here, many species (including sticklebacks) are known to transfer information chemically via eggs, e.g. hormonally mediated maternal effects (Mousseau and Fox 1998), that influence offspring behaviour, physiology and growth (papers listed in Ho and Burggren 2010), and can also be differentially provisioned depending on maternal environment (e.g. Giesing et al. 2011; Welch et al. 2014). Furthermore, maternal mRNA can be stabilized in the yolk and has been shown to act as a messenger in the embryo (Weeks and Melton 1987; Wang et al. 1998; Lin et al. 2000; Huttenhuis et al. 2006) Finally, epigenetic variation, e.g. differential gene expression across generations, is another potential mechanism underlying differences between acute versus developmental acclimation responses, and is discussed in detail below.

### Differential gene expression across generations

Our transcriptomic analyses clearly showed that thermal acclimation history had a profound effect on gene expression patterns by significantly changing transcript levels in >6% of the genome. Our factorial experimental design has the advantage that it can differentiate between up‐ and down‐regulation of genes depending on pure transgenerational effects (main effects of D and MGD), and mismatches in thermal history between generations (interaction effects). Interestingly, the immediate environment experienced by offspring had the smallest effect on differential gene expression, mainly influencing signalling, and phosphate and amino acid transporters. Maternal granddam environment, on the other hand, had a substantial influence on the expression patterns of genes, especially when maternal and grandmaternal temperatures differed from one another, highlighting the benefits of using factorial designs for transcriptomic studies. This pattern was particularly strong for several cytochrome P450 genes. Sticklebacks are often employed as indicator species in ecotoxicology with cytochrome P450 genes used as molecular markers for detoxification (Williams et al. 2009). Our results show that the environment of previous generations can also influence the expression of these marker genes, indicating that transgenerational effects should not be forgotten in environmental quality assessments relying on indicator species. Together with the strong up‐regulation of these genes when maternal and grandmaternal environments differed, this may suggest that stable thermal environments reduce stress arising from accumulation of metabolic waste. This also matches the strong up‐regulation of wounding/hemostasis genes in mismatching offspring and grandmother environments, as well as the general enrichment of grandmaternally imprinted genes involved in these processes.

The largest impact on metabolic processes stemmed from the thermal environment experienced by the mother, both in terms of numbers of differentially regulated genes and the number of biological processes they were associated with. The increasing importance of the environment experienced by previous generations shown here supports other recent findings of transgenerational acclimation effects on gene expression, while also describing a more complex picture of organismal responses (Veilleux et al. 2015; DeWit et al. *in press*). In general, the thermal environment interferes with several basal organismic processes including circadian rhythm (see maternal down‐regulation of *perp1b, cry1aa*; Podrabsky and Somero 2004) and stress response by heat shock proteins (Iwama et al. 1998). Surprisingly, other studies on transgenerational thermal acclimation did not find long‐term transcriptional responses involving immediate stress response genes (e.g. heat shock proteins like *HSP70*; Veilleux et al. 2015), indicating that long‐lasting temperature regime shifts did not impose chronic stress on the fish, but rather, shifted their metabolism to other steady states. We also did not observe an enrichment of genes involved in responses to heat stress. Still, while Veilleux et al. (2015) did not observe any significant regulation of heat shock proteins or other chaperones, we observed a substantial up‐regulation of *HSP90* and *HSP10*. Both genes are involved in ATP‐dependent protein folding (Hohfeld and Hartl 1994; Young et al. 2001; Korobeinikova et al. 2012), supporting our results of increased protein production in mitochondria. All mitochondria encoded genes showed higher transcript levels when mothers developed at elevated temperature. Higher transcript levels of mitochondrial genes was correlated with concomitant up‐regulation of several processes associated with gene expression in the mitochondria, indicating extensive transgenerational cross‐talk between nuclear and mitochondrial genes (Kotiadis et al. 2014). This involved all stages of RNA metabolism, from mRNA metabolism to protein translation by mitochondrial ribosome biogenesis and tRNA modification, leading to higher rates of mitochondrial protein production, and consequently, to higher respiration rates. This is in contrast with transgenerational acclimation to ocean acidification in copepods where RNA transcription was down‐regulated (DeWit et al. *in press*), and thermal transgenerational acclimation in tropical reef fish that did not show increased protein production, but rather down‐regulation of protein elongation factors (Veilleux et al. 2015). Taken together, these studies indicate that transcriptional responses affecting functionally different processes are fine‐tuned depending on particular environmental cues or even species‐specific responses.

A noticeable number of the maternally regulated metabolism genes were involved in catabolic processes mainly involving proteins (e.g. proteasomes), which matches the enrichment and up‐regulation of translation genes, ultimately resulting in a higher protein turnover. The induction of amino‐acid transporters depending on offspring temperature supports this, and might indicate that transgenerationally regulated biosynthesis may lead to higher substrate availability from increased protein turnover. The up‐regulation of the translation machinery we observed here seemed to be mainly targeted on the expression of mitochondrial genes, and ultimately energy production. While this pattern was consistently observed when mothers came from a 21°C environment, a mismatch between maternal and grandmaternal environment seemed to induce the transcription of ATP‐synthetase genes, indicating that environmental mismatches of previous generations can lead to higher or lower energy demands that are modulated by the grandmaternal thermal environment. The increased energy demand imposed by maternal thermal environment did not, however, lead to a putative metabolic shift towards certain substrates. In other species, such shifts were observed during developmental acclimation towards glucose metabolism (Windisch et al. 2014) or by transgenerational acclimation towards lipid metabolism (Veilleux et al. 2015). Genes involved in protein or lipid metabolism in sticklebacks were either up‐regulated (e.g. *acot8*,* pycr1b* or *ACSF3*) or down‐regulated (e.g. *hdc*,* acsf2* or *ACSL6*) by a 21°C maternal environment, indicating that either substrate could be converted for energy production. Transgenerational acclimation in sticklebacks, therefore, seemed to affect a wider range of metabolic processes, as reflected by their similar proportions in our enriched GO term lists. Directional enrichment was, rather, observed for more general terms like translation and transcription affecting mitochondrial energy production. To investigate the effect of differential substrate usage for energy production and organismic performance, phenotypic measurements need to be integrated into experimental approaches manipulating the available energy sources (Oellermann et al. 2012; Strobel et al. 2013).

### Linking transcriptomics to physiology

Our ultimate aim was to investigate how thermal acclimation history translates into mitochondrial respiratory capacity on the molecular transcriptional level. Here, we showed that offspring of mothers acclimated to elevated temperature had higher expression of all 13 mitochondria encoded genes. Although complex, gene up‐regulation and a higher numbers of transcripts can coincide with increased protein levels (but see Feder and Walser 2005; Pan et al. 2015), which could, in turn, entail higher respiration rates. Our transcriptomic and phenotypic mitochondrial respiration data seem to support this, as we found up‐regulation of genes involved in mitochondrial protein synthesis, suggesting that more proteins were actually produced. Also, while maternal environment had the strongest and most consistent effect on the expression of mitochondria encoded genes, transcript levels of both ATP‐synthetase genes differed depending on the mismatch between maternal and grandmaternal environments. Transcript levels were the lowest overall for offspring with a 21°C × 17°C grandmother‐mother acclimation history, and higher for offspring with a 17°C × 21°C thermal history, matching the pattern for MRCs in both groups. That transcript levels of the 21°C × 17°C group were even lower than those for offspring with a 17°C × 17°C thermal history further supports our tenet of lingering positive transgenerational effects of maternal grandmothers that experienced acute acclimation to 21°C. Developmental acclimation of mothers to 21°C, on the other hand, led to up‐regulation of both nuclear and mitochondrial genes involved in mitochondrial protein production, and likely to higher respiration rates. Taken together, our results demonstrate that acute or developmental acclimation to climate change cues during critical life stages of previous generations can lead to specific changes in gene expression of the nuclear and mitochondrial genomes that may result in optimized or more efficient mitochondrial function, with concomitant effects on offspring growth.

## Conclusions

The most direct consequence of global change for marine fish populations is likely to be physiological stress due to ocean warming, acidification and/or hypoxia (Crozier and Hutchings 2014). Yet, transgenerational effects can help to mediate some of these impacts (Munday 2014). Our study used a combined experimental approach that allowed us to gain a better understanding of the influence of acute versus developmental acclimation of previous generations on offspring phenotypes. By doing so, we can identify critical windows in the life history when organisms are most susceptible to environmental cues, and the consequences for future generations (Burton and Metcalfe 2014). Moreover, by investigating both gene expression profiles and mitochondrial respiratory capacities, we can start to build a link between molecular and cellular mechanisms potentially underlying transgenerational effects to ocean warming. To date, few studies have established such a link (but see Veilleux et al. 2015; DeWit et al. *in press*) despite this being essential to fully understand the ecological and evolutionary implications of transgenerational effects (Ho and Burggren 2010). Our study goes a step further because we can differentiate between the influence of persistent environmental change across generations and environmental mismatches between generations on multiple levels of biological complexity (molecular, cellular, whole organism), and so can begin to tease apart whether nongenetic inheritance effects accumulate or are reset with each generation (Shea et al. 2011; Herman et al. 2014).

Irrespective of the underlying mechanism(s), adaptive transgenerational effects could promote population persistence under climate change by allowing the mean phenotype to track a rapidly shifting optimum, however, such effects may also weaken selection on genetic variation and adaptation in the long‐term, posing a challenge for predictive capabilities (Bonduriansky et al. 2012). Advancements in our mechanistic understanding of transgenerational effects (Veilleux et al. 2015) open the door to measuring these effects in wild populations, and when combined with studies of genetic changes (DeWit et al. *in press*), can contribute greatly to our knowledge of the evolution of tolerance limits (Sunday et al. 2014). Ultimately, the ability to assess the contribution of transgenerational effects to variation in fitness among individuals will have clear implications for how we maintain biodiversity and evolutionary potential of natural and managed populations in a warming ocean.

## Data archiving

All raw Illumina reads deposited at European Nucleotide Archive (Study accession no. PRJEB12613). Mitochondrial respiration assay data deposited at PANGAEA (doi.org/10.1594/PANGAEA.857796).

## Supporting information


**Table S1.** Experimental design depicting the thermal acclimation history of third generation (F2) marine sticklebacks used in the respiration assays and transcriptomic analyses.

**Table S2.** Sequencing statistics including total reads per library, mapped reads per library, and reads mapped to mitochondrial genes.

**Table S3.** Enriched GO‐terms for all treatment levels and their interactions with number of enriched genes and the corresponding Fisher's exact test statistics.

**Table S4.** Genes representing enriched processes with their respective ensembl gene ID, name and description.
