# Supplementary material for: Transgenerational effects persist down the maternal line in marine sticklebacks: gene expression matches physiology in a warming ocean
Source: Evol Appl. 2016 Feb 28;9(9):1096–111. doi: 10.1111/eva.12370 (PMC5039323; doi:10.1111/eva.12370)
Supplement: Supplementary file 1 — Table S1. Experimental design depicting the thermal acclimation history of third generation (F2) marine sticklebacks used in the respiration assays and transcriptomic analyses. [file EVA-9-1096-s001.pdf]

Supplemental table 1. Crossing designs for *Gasterosteus aculeatus* (a) F1 adults used as parental fish, (b) F2 offspring families, and (c) F2 adults used in the respiration and transcriptome experiments grouped by maternal granddam (MGD), maternal (Dam) and offspring thermal history. In (a): F1 crosses are shown as male (grandsire) °C x female (granddam) °C (e.g. 17x17) reared at either 17°C or 21°C (parental temperature). F1 temperature combination groups are depicted as G1, G2, etc. In (b): F2 crosses between G groups were reared at 17°C and 21°C (21° C not shown). The number of F2 families produced in each GxG cross combination is indicated. In (c): Respiration R groups were produced by pooling up to 20 randomly selected adult fish from the corresponding F2 offspring GxG groups. GxG groups are shown as male x female.

(a) F1 adults

F0 Grandparental °C	F1 Parental °C	F1 Group
17x17	17	G1
17x21	17	G2
21x17	17	G3
21x21	17	G4
17x17	21	G5
17x21	21	G6
21x17	21	G7
21x21	21	G8

(b) F2 offspring

		Female							
		G1	G2	G3	G4	G5	G6	G7	G8
Male	G1	n=3							n=0
	G2		n=3					n=3	
	G3			n=2			n=4		
	G4		n=1		n=2	n=6			
	G5				n=2	n=3			
	G6			n=1			n=1		
	G7		n=4			n=3		n=1	
	G8	n=0							n=0

(c) F2 adults

		Thermal history (°C)			F2 offspring GxG groups				
Respiration groups	R1	MGD 17	Dam 17	Offspring 17	G1xG1	G3xG3	G6xG3		
	R2			Offspring 21	G1xG1	G3xG3	G6xG3		
	R3	MGD 17	Dam 21	Offspring 17	G4xG5	G5xG5	G7xG5	G2xG7	G7xG7
	R4			Offspring 21	G4xG5	G5xG5	G7xG5	G2xG7	G7xG7
	R5	MGD 21	Dam 17	Offspring 17	G2xG2	G4xG2	G7xG2	G4xG4	G5xG4
	R6			Offspring 21	G2xG2	G4xG2	G7xG2	G4xG4	G5xG4
	R7	MGD 21	Dam 21	Offspring 17	G3xG6	G6xG6			
	R8			Offspring 21	G3xG6	G6xG6			
