# Supplementary material for: Transgenerational effects persist down the maternal line in marine sticklebacks: gene expression matches physiology in a warming ocean
Source: Evol Appl. 2016 Feb 28;9(9):1096–111. doi: 10.1111/eva.12370 (PMC5039323; doi:10.1111/eva.12370)
Supplement: Supplementary file 2 — Table S2. Sequencing statistics including total reads per library, mapped reads per library, and reads mapped to mitochondrial genes. [file EVA-9-1096-s002.pdf]

A) Sequencing statistics

Library	group	reads	mapped to gene	mapped to mitochondria
R1_1B	GD17_D17_O	9481332	6073205	1074871
R1_2		6990593	4474250	900198
R1_3		6876100	4316331	737617
R1_4		7772242	4879880	662146
R1_5		5692752	3384134	327715
R1_6		5984048	3611099	662324
R2_1	GD17_D17_O	4697905	2756612	372263
R2_2		5198497	3183361	436388
R2_3		6517981	3980271	381884
R2_4		4491867	2726597	424166
R2_5B		4851279	2863020	607416
R2_6B		6067956	3762234	645258
R3_1	GD21_D17_O	5487763	3392055	520339
R3_2		5622223	3384716	719796
R3_3		4179089	2544805	533121
R3_4		4409082	2591539	390050
R3_5B		5994478	3429731	355293
R4_1	GD21_D17_O	6928838	4130637	285776
R4_2		9338066	5944483	966423
R4_3		6072587	3672546	616991
R4_4		5395632	3202813	564885
R4_5		5411184	3048390	326221
R4_6		6002291	3885449	832994
R5_1	GD17_D21_O	6753830	4451656	923805
R5_2		7211180	4613862	1184305
R5_3		6998082	4568383	1108032
R5_4		5526711	3398552	663669
R5_5		5382127	3346762	517322
R5_6		4480990	2672956	419460
R6_1	GD17_D21_O	5975516	3848173	673301
R6_2		6774229	4409762	775638
R6_3		5711891	3524042	629961
R6_4		5719137	3482818	497177
R6_5		5671683	3436515	599881
R6_6		5991996	3546831	744283
R7_1	GD21_D21_O	6044454	3495223	843643
R7_2		8581432	5254181	867010
R7_3		4760246	2886590	694952
R7_4		2922612	1690673	309346
R7_5		3685278	2170085	369900
R7_6		4832622	2904044	726851
	Sum	**242487801**	**148939266**	**25892671**
