# Supplementary material for: Transgenerational effects persist down the maternal line in marine sticklebacks: gene expression matches physiology in a warming ocean
Source: Evol Appl. 2016 Feb 28;9(9):1096–111. doi: 10.1111/eva.12370 (PMC5039323; doi:10.1111/eva.12370)
Supplement: Supplementary file 3 — Table S3. Enriched GO‐terms for all treatment levels and their interactions with number of enriched genes and the corresponding Fisher's exact test statistics. [file EVA-9-1096-s003.pdf]

Enriched GO terms

Group	GO.ID	Term	Annotated	Significant	Expected	Fisher	N
Offspring 17°C vs 21°	GO:0023021	termination of signal transduction	8	4	0.97	0.01	17
	GO:0038032	termination of G-protein coupled recepto...	8	4	0.97	0.01	
	GO:0045744	negative regulation of G-protein coupled...	8	4	0.97	0.01	
	GO:0042491	auditory receptor cell differentiation	5	3	0.61	0.015	
	GO:0060113	inner ear receptor cell differentiation	9	4	1.1	0.017	
	GO:0071705	nitrogen compound transport	14	5	1.71	0.02	
	GO:0014070	response to organic cyclic compound	35	9	4.26	0.021	
	GO:0042490	mechanoreceptor differentiation	10	4	1.22	0.025	
	GO:0015711	organic anion transport	15	5	1.83	0.028	
	GO:0006820	anion transport	31	8	3.78	0.028	
	GO:0007186	G-protein coupled receptor signaling pat...	68	14	8.28	0.031	
	GO:0071407	cellular response to organic cyclic comp...	32	8	3.9	0.034	
	GO:0008277	regulation of G-protein coupled receptor...	11	4	1.34	0.035	
	GO:0072006	nephron development	11	4	1.34	0.035	
	GO:0006631	fatty acid metabolic process	7	3	0.85	0.043	
	GO:0015849	organic acid transport	7	3	0.85	0.043	
	GO:0046942	carboxylic acid transport	7	3	0.85	0.043	
Dam 17°C vs 21°	GO:0044237	cellular metabolic process	2184	494	443.43	0.00032	92
	GO:0007623	circadian rhythm	9	7	1.83	0.00034	
	GO:0048511	rhythmic process	9	7	1.83	0.00034	
	GO:0034660	ncRNA metabolic process	96	34	19.49	0.00037	
	GO:0034470	ncRNA processing	46	19	9.34	0.0009	
	GO:0010467	gene expression	774	189	157.15	0.00149	
	GO:0044265	cellular macromolecule catabolic process	88	30	17.87	0.00164	
	GO:0044248	cellular catabolic process	135	41	27.41	0.00327	
	GO:0006402	mRNA catabolic process	9	6	1.83	0.0033	
	GO:0009056	catabolic process	189	54	38.37	0.0036	
	GO:0006399	tRNA metabolic process	73	25	14.82	0.00362	
	GO:0009057	macromolecule catabolic process	104	33	21.12	0.00373	
	GO:0044260	cellular macromolecule metabolic process	1737	390	352.68	0.00418	
	GO:0006364	rRNA processing	18	9	3.65	0.00466	
	GO:0022613	ribonucleoprotein complex biogenesis	31	13	6.29	0.00491	
	GO:0009059	macromolecule biosynthetic process	766	183	155.53	0.00504	
	GO:1901576	organic substance biosynthetic process	943	221	191.46	0.00538	
	GO:0034645	cellular macromolecule biosynthetic proc...	763	182	154.92	0.00553	
	GO:1901362	organic cyclic compound biosynthetic pro...	628	152	127.51	0.00642	
	GO:0006401	RNA catabolic process	10	6	2.03	0.00684	
	GO:0044249	cellular biosynthetic process	920	215	186.79	0.00698	
	GO:0032922	circadian regulation of gene expression	5	4	1.02	0.00709	
	GO:0034308	primary alcohol metabolic process	5	4	1.02	0.00709	
	GO:0016072	rRNA metabolic process	19	9	3.86	0.00728	
	GO:0019438	aromatic compound biosynthetic process	603	146	122.43	0.00744	
	GO:0030163	protein catabolic process	85	27	17.26	0.00813	
	GO:0080090	regulation of primary metabolic process	514	126	104.36	0.00837	
	GO:0044271	cellular nitrogen compound biosynthetic ...	610	147	123.85	0.00865	
	GO:0032774	RNA biosynthetic process	501	123	101.72	0.00869	
	GO:0071704	organic substance metabolic process	2542	552	516.12	0.00887	
	GO:0018130	heterocycle biosynthetic process	615	148	124.87	0.00888	
	GO:1901575	organic substance catabolic process	176	49	35.73	0.00919	
	GO:0034654	nucleobase-containing compound biosynthe	571	138	115.93	0.00983	
	GO:0019219	regulation of nucleobase-containing comp...	458	113	92.99	0.01004	
	GO:0006351	transcription, DNA-templated	499	122	101.32	0.01028	
	GO:0097659	nucleic acid-templated transcription	499	122	101.32	0.01028	
	GO:0051171	regulation of nitrogen compound metaboli...	459	113	93.19	0.01075	
	GO:0060113	inner ear receptor cell differentiation	20	9	4.06	0.0109	
	GO:0009058	biosynthetic process	982	226	199.38	0.01183	
	GO:0051252	regulation of RNA metabolic process	452	111	91.77	0.01233	
	GO:0044238	primary metabolic process	2455	532	498.46	0.01319	
	GO:0031323	regulation of cellular metabolic process	523	126	106.19	0.01486	
	GO:0009889	regulation of biosynthetic process	461	112	93.6	0.01653	
	GO:0060255	regulation of macromolecule metabolic pr...	534	128	108.42	0.01663	
	GO:0016071	mRNA metabolic process	54	18	10.96	0.01687	
	GO:0031326	regulation of cellular biosynthetic proc...	457	111	92.79	0.01711	
	GO:0000956	nuclear-transcribed mRNA catabolic proce...	6	4	1.22	0.01785	
	GO:0006835	dicarboxylic acid transport	6	4	1.22	0.01785	
	GO:0010498	proteasomal protein catabolic process	6	4	1.22	0.01785	
	GO:0043161	proteasome-mediated ubiquitin-dependent	6	4	1.22	0.01785	
	GO:0006511	ubiquitin-dependent protein catabolic pr...	66	21	13.4	0.01796	
	GO:0019941	modification-dependent protein catabolic...	66	21	13.4	0.01796	
	GO:0043632	modification-dependent macromolecule cat.	66	21	13.4	0.01796	
	GO:2000112	regulation of cellular macromolecule bio...	454	110	92.18	0.01887	
	GO:0042254	ribosome biogenesis	25	10	5.08	0.01896	
	GO:0006355	regulation of transcription, DNA-templat...	441	107	89.54	0.01963	
	GO:1903506	regulation of nucleic acid-templated tra...	441	107	89.54	0.01963	
	GO:2001141	regulation of RNA biosynthetic process	441	107	89.54	0.01963	
	GO:0006457	protein folding	51	17	10.35	0.0199	
	GO:0010556	regulation of macromolecule biosynthetic...	455	110	92.38	0.02009	
	GO:0071826	ribonucleoprotein complex subunit organi...	12	6	2.44	0.02073	
	GO:0090101	negative regulation of transmembrane rec...	12	6	2.44	0.02073	
	GO:0048794	swim bladder development	9	5	1.83	0.02077	
	GO:0071542	dopaminergic neuron differentiation	9	5	1.83	0.02077	
	GO:0044257	cellular protein catabolic process	75	23	15.23	0.02138	
	GO:0051603	proteolysis involved in cellular protein...	75	23	15.23	0.02138	
	GO:0010468	regulation of gene expression	486	116	98.68	0.02497	
	GO:0006066	alcohol metabolic process	16	7	3.25	0.02861	
	GO:0060119	inner ear receptor cell development	16	7	3.25	0.02861	
	GO:0019222	regulation of metabolic process	576	135	116.95	0.02876	
	GO:0008152	metabolic process	3055	649	620.28	0.02877	
	GO:1901564	organonitrogen compound metabolic proces	280	70	56.85	0.02899	
	GO:0043170	macromolecule metabolic process	2026	439	411.35	0.03012	
	GO:0042491	auditory receptor cell differentiation	13	6	2.64	0.03202	
	GO:0008033	tRNA processing	27	10	5.48	0.03314	
	GO:0051235	maintenance of location	10	5	2.03	0.03471	
	GO:0006023	aminoglycan biosynthetic process	7	4	1.42	0.03502	
	GO:0021591	ventricular system development	7	4	1.42	0.03502	
	GO:0043401	steroid hormone mediated signaling pathw..	39	13	7.92	0.03908	
	GO:0006520	cellular amino acid metabolic process	117	32	23.76	0.03941	
	GO:0014070	response to organic cyclic compound	71	21	14.42	0.03968	
	GO:0009451	RNA modification	17	7	3.45	0.04034	
	GO:0009948	anterior/posterior axis specification	17	7	3.45	0.04034	
	GO:0043038	amino acid activation	47	15	9.54	0.04061	
	GO:0043039	tRNA aminoacylation	47	15	9.54	0.04061	
	GO:0015711	organic anion transport	28	10	5.69	0.04246	
	GO:0045934	negative regulation of nucleobase-contai...	28	10	5.69	0.04246	
	GO:0051172	negative regulation of nitrogen compound...	28	10	5.69	0.04246	
	GO:0006221	pyrimidine nucleotide biosynthetic proce...	14	6	2.84	0.04664	
	GO:0031329	regulation of cellular catabolic process	14	6	2.84	0.04664	
	GO:0071407	cellular response to organic cyclic comp...	64	19	12.99	0.04713	
	GO:0009755	hormone-mediated signaling pathway	40	13	8.12	0.04752	
21°C	GO:0010033	response to organic substance	71	20	9.01	0.00034	53
	GO:0007599	hemostasis	11	6	1.4	0.00106	
	GO:0055123	digestive system development	48	14	6.09	0.00181	
	GO:0042221	response to chemical	137	29	17.38	0.00305	
	GO:0071495	cellular response to endogenous stimulus	46	13	5.84	0.00355	
	GO:0071310	cellular response to organic substance	57	15	7.23	0.00382	
	GO:0070887	cellular response to chemical stimulus	74	18	9.39	0.00405	
	GO:0007596	blood coagulation	10	5	1.27	0.00468	
	GO:0050878	regulation of body fluid levels	14	6	1.78	0.00493	
	GO:0033993	response to lipid	38	11	4.82	0.00585	
	GO:0071907	determination of digestive tract left/ri...	7	4	0.89	0.00652	
	GO:0009719	response to endogenous stimulus	55	14	6.98	0.00705	
	GO:0071383	cellular response to steroid hormone sti...	34	10	4.31	0.00751	
	GO:0071396	cellular response to lipid	34	10	4.31	0.00751	
	GO:0050817	coagulation	11	5	1.4	0.0077	
	GO:0032870	cellular response to hormone stimulus	35	10	4.44	0.00935	
	GO:0048545	response to steroid hormone	36	10	4.57	0.01153	
	GO:0000280	nuclear division	8	4	1.01	0.01175	
	GO:0042981	regulation of apoptotic process	53	13	6.72	0.01278	
	GO:0009725	response to hormone	37	10	4.69	0.01406	
	GO:0048565	digestive tract development	37	10	4.69	0.01406	
	GO:0071407	cellular response to organic cyclic comp...	37	10	4.69	0.01406	
	GO:0048916	posterior lateral line development	17	6	2.16	0.01456	
	GO:0010941	regulation of cell death	54	13	6.85	0.01495	
	GO:0043067	regulation of programmed cell death	54	13	6.85	0.01495	

Group	GO.ID	Term	Annotated	Significant	Expected	Fisher	N
Cluster I	GO:0010830	regulation of myotube differentiation	2	1	0.02	0.024	31
	GO:0030240	skeletal muscle thin filament assembly	2	1	0.02	0.024	
	GO:0048742	regulation of skeletal muscle fiber deve...	2	1	0.02	0.024	
	GO:0048769	sarcomerogenesis	2	1	0.02	0.024	
	GO:0051147	regulation of muscle cell differentiatio...	2	1	0.02	0.024	
	GO:0051153	regulation of striated muscle cell diffe...	2	1	0.02	0.024	
	GO:0055081	anion homeostasis	2	1	0.02	0.024	
	GO:0060561	apoptotic process involved in morphogene...	2	1	0.02	0.024	
	GO:1902742	apoptotic process involved in developmen...	2	1	0.02	0.024	
	GO:0043623	cellular protein complex assembly	21	2	0.26	0.026	
	GO:0046488	phosphatidylinositol metabolic process	21	2	0.26	0.026	
	GO:0071705	nitrogen compound transport	21	2	0.26	0.026	
	GO:0006650	glycerophospholipid metabolic process	22	2	0.27	0.029	
	GO:0046486	glycerolipid metabolic process	22	2	0.27	0.029	
	GO:0010623	developmental programmed cell death	3	1	0.04	0.036	
	GO:0014034	neural crest cell fate commitment	3	1	0.04	0.036	
	GO:0014036	neural crest cell fate specification	3	1	0.04	0.036	
	GO:0016202	regulation of striated muscle tissue dev...	3	1	0.04	0.036	
	GO:0030241	skeletal muscle myosin thick filament as...	3	1	0.04	0.036	
	GO:0031033	myosin filament organization	3	1	0.04	0.036	
	GO:0031034	myosin filament assembly	3	1	0.04	0.036	
	GO:0046834	lipid phosphorylation	3	1	0.04	0.036	
	GO:0046854	phosphatidylinositol phosphorylation	3	1	0.04	0.036	
	GO:0048641	regulation of skeletal muscle tissue dev...	3	1	0.04	0.036	
	GO:0071688	striated muscle myosin thick filament as...	3	1	0.04	0.036	
	GO:1901861	regulation of muscle tissue development	3	1	0.04	0.036	
	GO:0034622	cellular macromolecular complex assembly	29	2	0.35	0.048	
	GO:0003094	glomerular filtration	4	1	0.05	0.048	
	GO:0007172	signal complex assembly	4	1	0.05	0.048	
	GO:0015696	ammonium transport	4	1	0.05	0.048	
	GO:0048634	regulation of muscle organ development	4	1	0.05	0.048	
Cluster II	GO:0031058	positive regulation of histone modificat...	3	2	0.06	0.0011	82
	GO:0048845	venous blood vessel morphogenesis	3	2	0.06	0.0011	
	GO:1903310	positive regulation of chromatin modific...	3	2	0.06	0.0011	
	GO:2001252	positive regulation of chromosome organi...	3	2	0.06	0.0011	
	GO:0032270	positive regulation of cellular protein ...	13	3	0.25	0.0018	
	GO:0051247	positive regulation of protein metabolic...	13	3	0.25	0.0018	
	GO:0090124	N-4 methylation of cytosine	4	2	0.08	0.0022	
	GO:0060855	venous endothelial cell migration involv...	5	2	0.1	0.0036	
	GO:0031401	positive regulation of protein modificat...	6	2	0.12	0.0054	
	GO:0060841	venous blood vessel development	7	2	0.14	0.0075	
	GO:0055007	cardiac muscle cell differentiation	21	3	0.41	0.0075	
	GO:0031056	regulation of histone modification	8	2	0.16	0.0098	
	GO:0032776	DNA methylation on cytosine	8	2	0.16	0.0098	
	GO:1902275	regulation of chromatin organization	8	2	0.16	0.0098	
	GO:1903308	regulation of chromatin modification	8	2	0.16	0.0098	
	GO:0060485	mesenchyme development	94	6	1.84	0.0099	
	GO:0014032	neural crest cell development	45	4	0.88	0.0111	
	GO:0033044	regulation of chromosome organization	9	2	0.18	0.0125	
	GO:0016477	cell migration	198	9	3.87	0.015	
	GO:0007264	small GTPase mediated signal transductio...	304	12	5.94	0.0151	
	GO:0008284	positive regulation of cell proliferatio...	10	2	0.2	0.0154	
	GO:0014033	neural crest cell differentiation	50	4	0.98	0.016	
	GO:0048870	cell motility	201	9	3.93	0.0164	
	GO:0051674	localization of cell	201	9	3.93	0.0164	
	GO:0050789	regulation of biological process	1974	50	38.57	0.0166	
	GO:0010638	positive regulation of organelle organiz...	11	2	0.21	0.0186	
	GO:0001569	patterning of blood vessels	12	2	0.23	0.022	
	GO:0048754	branching morphogenesis of an epithelial...	12	2	0.23	0.022	
	GO:0035051	cardiocyte differentiation	32	3	0.63	0.0239	
	GO:0061138	morphogenesis of a branching epithelium	13	2	0.25	0.0257	
	GO:1901136	carbohydrate derivative catabolic proces...	13	2	0.25	0.0257	
	GO:0014031	mesenchymal cell development	59	4	1.15	0.0276	
	GO:0001667	ameboidal-type cell migration	120	6	2.34	0.0295	
	GO:0035023	regulation of Rho protein signal transdu...	89	5	1.74	0.0296	
	GO:0001755	neural crest cell migration	35	3	0.68	0.0302	
	GO:0048864	stem cell development	61	4	1.19	0.0308	
	GO:0065007	biological regulation	2090	51	40.83	0.0311	
	GO:0007266	Rho protein signal transduction	92	5	1.8	0.0335	
	GO:0048762	mesenchymal cell differentiation	64	4	1.25	0.0358	
	GO:0048738	cardiac muscle tissue development	38	3	0.74	0.0374	
	GO:0000724	double-strand break repair via homologou...	2	1	0.04	0.0387	
	GO:0000725	recombinational repair	2	1	0.04	0.0387	
	GO:0001936	regulation of endothelial cell prolifera...	2	1	0.04	0.0387	
	GO:0001938	positive regulation of endothelial cell ...	2	1	0.04	0.0387	
	GO:0002159	desmosome assembly	2	1	0.04	0.0387	
	GO:0003190	atrioventricular valve formation	2	1	0.04	0.0387	
	GO:0003342	proepicardium development	2	1	0.04	0.0387	
	GO:0003343	septum transversum development	2	1	0.04	0.0387	
	GO:0006473	protein acetylation	2	1	0.04	0.0387	
	GO:0006475	internal protein amino acid acetylation	2	1	0.04	0.0387	
	GO:0008612	peptidyl-lysine modification to peptidyl...	2	1	0.04	0.0387	
	GO:0009411	response to UV	2	1	0.04	0.0387	
	GO:0016573	histone acetylation	2	1	0.04	0.0387	
	GO:0018393	internal peptidyl-lysine acetylation	2	1	0.04	0.0387	
	GO:0018394	peptidyl-lysine acetylation	2	1	0.04	0.0387	
	GO:0021755	eurydendroid cell differentiation	2	1	0.04	0.0387	
	GO:0033278	cell proliferation in midbrain	2	1	0.04	0.0387	
	GO:0035065	regulation of histone acetylation	2	1	0.04	0.0387	
	GO:0035066	positive regulation of histone acetylati...	2	1	0.04	0.0387	
	GO:0048771	tissue remodeling	2	1	0.04	0.0387	
	GO:0050679	positive regulation of epithelial cell p...	2	1	0.04	0.0387	
	GO:0060174	limb bud formation	2	1	0.04	0.0387	
	GO:0060465	pharynx development	2	1	0.04	0.0387	
	GO:0061323	cell proliferation involved in heart mor...	2	1	0.04	0.0387	
	GO:1901983	regulation of protein acetylation	2	1	0.04	0.0387	
	GO:1901985	positive regulation of protein acetylati...	2	1	0.04	0.0387	
	GO:2000136	regulation of cell proliferation involve...	2	1	0.04	0.0387	
	GO:2000756	regulation of peptidyl-lysine acetylatio...	2	1	0.04	0.0387	
	GO:2000758	positive regulation of peptidyl-lysine a...	2	1	0.04	0.0387	
	GO:0030902	hindbrain development	66	4	1.29	0.0395	
	GO:0044763	single-organism cellular process	3022	69	59.04	0.0398	
	GO:0050794	regulation of cellular process	1878	46	36.69	0.0406	
	GO:0018205	peptidyl-lysine modification	17	2	0.33	0.0425	
	GO:0042981	regulation of apoptotic process	100	5	1.95	0.0454	
	GO:0043067	regulation of programmed cell death	101	5	1.97	0.047	
	GO:0006304	DNA modification	18	2	0.35	0.0472	
	GO:0006305	DNA alkylation	18	2	0.35	0.0472	
	GO:0006306	DNA methylation	18	2	0.35	0.0472	
	GO:0043534	blood vessel endothelial cell migration	18	2	0.35	0.0472	
	GO:0044728	DNA methylation or demethylation	18	2	0.35	0.0472	
	GO:0031325	positive regulation of cellular metaboli...	70	4	1.37	0.0474	
	GO:0018193	peptidyl-amino acid modification	42	3	0.82	0.0481	
	GO:0006364	rRNA processing	23	6	1.12	0.00062	71
	GO:0009451	RNA modification	23	6	1.12	0.00062	
	GO:0016072	rRNA metabolic process	24	6	1.16	0.0008	
	GO:1901575	organic substance catabolic process	213	21	10.33	0.00146	
	GO:0009056	catabolic process	228	22	11.06	0.0015	
	GO:0001895	retina homeostasis	12	4	0.58	0.00197	
	GO:0045494	photoreceptor cell maintenance	12	4	0.58	0.00197	
	GO:0044265	cellular macromolecule catabolic process	109	13	5.29	0.00224	
	GO:0006429	leucyl-tRNA aminoacylation	2	2	0.1	0.00235	
	GO:0044248	cellular catabolic process	165	17	8	0.00255	
	GO:0008033	tRNA processing	30	6	1.46	0.00275	
	GO:0006418	tRNA aminoacylation for protein translat...	53	8	2.57	0.00368	
	GO:0014823	response to activity	14	4	0.68	0.00369	
	GO:0044237	cellular metabolic process	2631	151	127.62	0.00401	
	GO:0043038	amino acid activation	54	8	2.62	0.00415	
	GO:0043039	tRNA aminoacylation	54	8	2.62	0.00415	
	GO:0006082	organic acid metabolic process	233	21	11.3	0.00433	
	GO:0043436	oxoacid metabolic process	233	21	11.3	0.00433	
	GO:0006511	ubiquitin-dependent protein catabolic pr...	82	10	3.98	0.00599	
	GO:0019941	modification-dependent protein catabolic...	82	10	3.98	0.00599	
	GO:0043632	modification-dependent macromolecule cat...	82	10	3.98	0.00599	

MGD 17°C vs .	GO:0043401 steroid hormone mediated signaling pathw..	22	7	2.79	0.01556	
	GO:0035469 determination of pancreatic left/right a...	5	3	0.63	0.01664	
	GO:0046686 response to cadmium ion	5	3	0.63	0.01664	
	GO:0014070 response to organic cyclic compound	44	11	5.58	0.01847	
	GO:0006304 DNA modification	9	4	1.14	0.01906	
	GO:0006305 DNA alkylation	9	4	1.14	0.01906	
	GO:0006306 DNA methylation	9	4	1.14	0.01906	
	GO:0044728 DNA methylation or demethylation	9	4	1.14	0.01906	
	GO:0009755 hormone-mediated signaling pathway	23	7	2.92	0.01999	
	GO:0006915 apoptotic process	68	15	8.63	0.0206	
	GO:0012501 programmed cell death	69	15	8.75	0.02337	
	GO:0031016 pancreas development	40	10	5.07	0.02417	
	GO:0008219 cell death	70	15	8.88	0.02642	
	GO:0016265 death	70	15	8.88	0.02642	
	GO:0048285 organelle fission	10	4	1.27	0.02864	
	GO:0007067 mitotic nuclear division	6	3	0.76	0.03018	
	GO:0031056 regulation of histone modification	6	3	0.76	0.03018	
	GO:0033044 regulation of chromosome organization	6	3	0.76	0.03018	
	GO:0060841 venous blood vessel development	6	3	0.76	0.03018	
	GO:1902275 regulation of chromatin organization	6	3	0.76	0.03018	
	GO:1903308 regulation of chromatin modification	6	3	0.76	0.03018	
	GO:0001708 cell fate specification	20	6	2.54	0.0328	
	GO:0070121 Kupffer's vesicle development	20	6	2.54	0.0328	
	GO:0044707 single-multicellular organism process	908	131	115.19	0.03749	
	GO:0048881 mechanosensory lateral line system devel...	21	6	2.66	0.04117	
	GO:0048915 posterior lateral line system developmen...	21	6	2.66	0.04117	
	GO:0032776 DNA methylation on cytosine	7	3	0.89	0.04793	
	GO:0007420 brain development	108	20	13.7	0.04925	
OxD	GO:0006163 purine nucleotide metabolic process	5	3	0.46	0.0061	8
	GO:0009117 nucleotide metabolic process	5	3	0.46	0.0061	
	GO:0072521 purine-containing compound metabolic pro.	5	3	0.46	0.0061	
	GO:0006753 nucleoside phosphate metabolic process	6	3	0.55	0.0114	
	GO:0055086 nucleobase-containing small molecule met...	6	3	0.55	0.0114	
	GO:0044281 small molecule metabolic process	18	5	1.66	0.0162	
	GO:0019637 organophosphate metabolic process	8	3	0.74	0.0283	
	GO:0044710 single-organism metabolic process	39	7	3.59	0.0475	
OxMGD	GO:0044765 single-organism transport	79	14	7.61	0.013	6
	GO:0055085 transmembrane transport	43	9	4.14	0.017	
	GO:1902578 single-organism localization	82	14	7.89	0.018	
	GO:0007422 peripheral nervous system development	7	3	0.67	0.023	
	GO:0006508 proteolysis	47	9	4.53	0.029	
	GO:0034220 ion transmembrane transport	8	3	0.77	0.034	
DxMGD	GO:0034660 ncRNA metabolic process	48	15	5.64	0.00023	43
	GO:0006399 tRNA metabolic process	38	12	4.46	0.00088	
	GO:0016070 RNA metabolic process	295	52	34.65	0.00096	
	GO:0006418 tRNA aminoacylation for protein translat...	27	9	3.17	0.00257	
	GO:0043038 amino acid activation	28	9	3.29	0.00341	
	GO:0043039 tRNA aminoacylation	28	9	3.29	0.00341	
	GO:0090304 nucleic acid metabolic process	352	57	41.34	0.00435	
	GO:0006364 rRNA processing	7	4	0.82	0.00489	
	GO:0042221 response to chemical	102	21	11.98	0.00611	
	GO:0009410 response to xenobiotic stimulus	16	6	1.88	0.00716	
	GO:0007498 mesoderm development	21	7	2.47	0.00774	
	GO:0016072 rRNA metabolic process	8	4	0.94	0.00888	
	GO:0034470 ncRNA processing	17	6	2	0.01	
	GO:1903047 mitotic cell cycle process	13	5	1.53	0.01251	
	GO:0007088 regulation of mitotic nuclear division	5	3	0.59	0.01338	
	GO:0048794 swim bladder development	5	3	0.59	0.01338	
	GO:0051783 regulation of nuclear division	5	3	0.59	0.01338	
	GO:0000278 mitotic cell cycle	18	6	2.11	0.01356	
	GO:0070887 cellular response to chemical stimulus	64	14	7.52	0.01402	
	GO:0006139 nucleobase-containing compound metabolic	404	61	47.45	0.01549	
	GO:0010033 response to organic substance	59	13	6.93	0.01663	
	GO:0071466 cellular response to xenobiotic stimulus	14	5	1.64	0.01762	
	GO:0006725 cellular aromatic compound metabolic pro...	430	64	50.51	0.01786	
	GO:0034641 cellular nitrogen compound metabolic pro...	432	64	50.74	0.01971	
	GO:0060485 mesenchyme development	37	9	4.35	0.02372	
	GO:0001895 retina homeostasis	6	3	0.7	0.02446	
	GO:0003140 determination of left/right asymmetry in...	6	3	0.7	0.02446	
	GO:0007346 regulation of mitotic cell cycle	6	3	0.7	0.02446	
	GO:0010564 regulation of cell cycle process	6	3	0.7	0.02446	
	GO:0045494 photoreceptor cell maintenance	6	3	0.7	0.02446	
	GO:0048368 lateral mesoderm development	6	3	0.7	0.02446	
	GO:0048919 posterior lateral line neuromast develop...	6	3	0.7	0.02446	
	GO:0046483 heterocycle metabolic process	432	63	50.74	0.02853	
	GO:0048916 posterior lateral line development	11	4	1.29	0.03145	
	GO:0070121 Kupffer's vesicle development	11	4	1.29	0.03145	
	GO:0006807 nitrogen compound metabolic process	475	68	55.79	0.03354	
	GO:1901360 organic cyclic compound metabolic proces...	444	64	52.15	0.03437	
	GO:0007067 mitotic nuclear division	7	3	0.82	0.03913	
	GO:0051302 regulation of cell division	7	3	0.82	0.03913	
	GO:0022402 cell cycle process	17	5	2	0.04058	
	GO:0022613 ribonucleoprotein complex biogenesis	17	5	2	0.04058	
	GO:0071310 cellular response to organic substance	47	10	5.52	0.04211	
	GO:0014070 response to organic cyclic compound	35	8	4.11	0.04533	

Cluster III	GO:0019752 carboxylic acid metabolic process	226	20	10.96	0.00649	
	GO:0042254 ribosome biogenesis	36	6	1.75	0.00705	
	GO:0001894 tissue homeostasis	26	5	1.26	0.00738	
	GO:0009057 macromolecule catabolic process	128	13	6.21	0.00888	
	GO:0048871 multicellular organismal homeostasis	28	5	1.36	0.01018	
	GO:0006402 mRNA catabolic process	10	3	0.49	0.01052	
	GO:0055085 transmembrane transport	351	27	17.03	0.01113	
	GO:0001743 optic placode formation	4	2	0.19	0.01319	
	GO:0021529 spinal cord oligodendrocyte cell differe...	4	2	0.19	0.01319	
	GO:0060249 anatomical structure homeostasis	41	6	1.99	0.0133	
	GO:0044281 small molecule metabolic process	407	30	19.74	0.01351	
	GO:0006401 RNA catabolic process	11	3	0.53	0.01395	
	GO:0071697 ectodermal placode morphogenesis	20	4	0.97	0.0142	
	GO:0022613 ribonucleoprotein complex biogenesis	42	6	2.04	0.0149	
	GO:0044257 cellular protein catabolic process	94	10	4.56	0.0152	
	GO:0051603 proteolysis involved in cellular protein...	94	10	4.56	0.0152	
	GO:0034655 nucleobase-containing compound catabolic...	22	4	1.07	0.01987	
	GO:0010996 response to auditory stimulus	5	2	0.24	0.02128	
	GO:0071699 olfactory placode morphogenesis	5	2	0.24	0.02128	
	GO:2001020 regulation of response to DNA damage sti...	5	2	0.24	0.02128	
	GO:0051235 maintenance of location	13	3	0.63	0.0225	
	GO:0044260 cellular macromolecule metabolic process	2104	119	102.06	0.02281	
	GO:0071696 ectodermal placode development	23	4	1.12	0.02316	
	GO:0090304 nucleic acid metabolic process	988	61	47.92	0.0243	
	GO:0006139 nucleobase-containing compound metabolic..	1144	69	55.49	0.02685	
	GO:0016070 RNA metabolic process	833	52	40.41	0.0309	
	GO:0010498 proteasomal protein catabolic process	6	2	0.29	0.0309	
	GO:0018149 peptide cross-linking	6	2	0.29	0.0309	
	GO:0043161 proteasome-mediated ubiquitin-dependent ..	6	2	0.29	0.0309	
	GO:0050885 neuromuscular process controlling balanc...	6	2	0.29	0.0309	
	GO:0060897 neural plate regionalization	6	2	0.29	0.0309	
	GO:0006790 sulfur compound metabolic process	37	5	1.79	0.0317	
	GO:0032259 methylation	63	7	3.06	0.03186	
	GO:0006412 translation	200	16	9.7	0.03305	
	GO:0090101 negative regulation of transmembrane rec...	15	3	0.73	0.03333	
	GO:0030163 protein catabolic process	107	10	5.19	0.03413	
	GO:0044712 single-organism catabolic process	78	8	3.78	0.03443	
	GO:0006520 cellular amino acid metabolic process	140	12	6.79	0.03866	
	GO:0000413 protein peptidyl-prolyl isomerization	16	3	0.78	0.03958	
	GO:0018208 peptidyl-proline modification	16	3	0.78	0.03958	
	GO:0031929 TOR signaling	7	2	0.34	0.04189	
	GO:0032006 regulation of TOR signaling	7	2	0.34	0.04189	
	GO:0060896 neural plate pattern specification	7	2	0.34	0.04189	
	GO:0009968 negative regulation of signal transducti...	111	10	5.38	0.04232	
	GO:0034641 cellular nitrogen compound metabolic pro...	1211	71	58.74	0.04333	
	GO:0007268 synaptic transmission	17	3	0.82	0.04639	
	GO:0060788 ectodermal placode formation	17	3	0.82	0.04639	
	GO:0010648 negative regulation of cell communicatio...	113	10	5.48	0.04688	
	GO:0023057 negative regulation of signaling	113	10	5.48	0.04688	
	GO:0006807 nitrogen compound metabolic process	1313	76	63.69	0.04732	

Cluster IV	GO:0014070 response to organic cyclic compound	73	7	1.67	0.0013	59
	GO:0010033 response to organic substance	130	9	2.97	0.0028	
	GO:0071407 cellular response to organic cyclic comp...	66	6	1.51	0.0038	
	GO:0007156 homophilic cell adhesion via plasma memb...	49	5	1.12	0.0049	
	GO:0098609 cell-cell adhesion	49	5	1.12	0.0049	
	GO:0098742 cell-cell adhesion via plasma-membrane a...	49	5	1.12	0.0049	
	GO:0070887 cellular response to chemical stimulus	142	9	3.25	0.005	
	GO:0009410 response to xenobiotic stimulus	32	4	0.73	0.0057	
	GO:0043363 nucleate erythrocyte differentiation	6	2	0.14	0.0073	
	GO:0042221 response to chemical	249	12	5.69	0.0111	
	GO:0071310 cellular response to organic substance	108	7	2.47	0.0114	
	GO:0009719 response to endogenous stimulus	91	6	2.08	0.0174	
	GO:1901700 response to oxygen-containing compound	25	3	0.57	0.0186	
	GO:1901698 response to nitrogen compound	10	2	0.23	0.0207	
	GO:0071466 cellular response to xenobiotic stimulus	27	3	0.62	0.0229	
	GO:0033993 response to lipid	72	5	1.65	0.0239	
	GO:0006790 sulfur compound metabolic process	28	3	0.64	0.0252	
	GO:0045926 negative regulation of growth	13	2	0.3	0.0343	
	GO:0071495 cellular response to endogenous stimulus	81	5	1.85	0.0372	
	GO:0006222 UMP biosynthetic process	2	1	0.05	0.0452	
	GO:0006275 regulation of DNA replication	2	1	0.05	0.0452	
	GO:0006534 cysteine metabolic process	2	1	0.05	0.0452	
	GO:0006787 porphyrin-containing compound catabolic ...	2	1	0.05	0.0452	
	GO:0006788 heme oxidation	2	1	0.05	0.0452	
	GO:0006826 iron ion transport	2	1	0.05	0.0452	
	GO:0008631 intrinsic apoptotic signaling pathway in...	2	1	0.05	0.0452	
	GO:0009173 pyrimidine ribonucleoside monophosphate ...	2	1	0.05	0.0452	
	GO:0009174 pyrimidine ribonucleoside monophosphate ...	2	1	0.05	0.0452	
	GO:0010039 response to iron ion	2	1	0.05	0.0452	
	GO:0014074 response to purine-containing compound	2	1	0.05	0.0452	
	GO:0021670 lateral ventricle development	2	1	0.05	0.0452	
	GO:0021678 third ventricle development	2	1	0.05	0.0452	
	GO:0032355 response to estradiol	2	1	0.05	0.0452	
	GO:0033015 tetrapyrrole catabolic process	2	1	0.05	0.0452	
	GO:0033198 response to ATP	2	1	0.05	0.0452	
	GO:0034599 cellular response to oxidative stress	2	1	0.05	0.0452	
	GO:0034755 iron ion transmembrane transport	2	1	0.05	0.0452	
	GO:0035046 pronuclear migration	2	1	0.05	0.0452	
	GO:0035124 embryonic caudal fin morphogenesis	2	1	0.05	0.0452	
	GO:0035264 multicellular organism growth	2	1	0.05	0.0452	
	GO:0036473 cell death in response to oxidative stre...	2	1	0.05	0.0452	
	GO:0040009 regulation of growth rate	2	1	0.05	0.0452	
	GO:0040014 regulation of multicellular organism gro...	2	1	0.05	0.0452	
	GO:0045843 negative regulation of striated muscle t...	2	1	0.05	0.0452	
	GO:0045967 negative regulation of growth rate	2	1	0.05	0.0452	
	GO:0046049 UMP metabolic process	2	1	0.05	0.0452	
	GO:0046683 response to organophosphorus	2	1	0.05	0.0452	
	GO:0048785 hatching gland development	2	1	0.05	0.0452	
	GO:0051984 positive regulation of chromosome segreg...	2	1	0.05	0.0452	
	GO:1900407 regulation of cellular response to oxida...	2	1	0.05	0.0452	
	GO:1900408 negative regulation of cellular response...	2	1	0.05	0.0452	
	GO:1901862 negative regulation of muscle tissue dev...	2	1	0.05	0.0452	
	GO:1902175 regulation of oxidative stress-induced i...	2	1	0.05	0.0452	
	GO:1902176 negative regulation of oxidative stress-...	2	1	0.05	0.0452	
	GO:1902883 negative regulation of response to oxida...	2	1	0.05	0.0452	
	GO:1903201 regulation of oxidative stress-induced c...	2	1	0.05	0.0452	
	GO:1903202 negative regulation of oxidative stress-...	2	1	0.05	0.0452	
	GO:0006811 ion transport	272	11	6.22	0.046	
	GO:0071383 cellular response to steroid hormone sti...	60	4	1.37	0.0476	

	GO:0050789 regulation of biological process	2234	141	109.32	0.00013	98
	GO:0050794 regulation of cellular process	2125	135	103.99	0.00015	
	GO:0065007 biological regulation	2365	146	115.73	0.00029	
	GO:0007623 circadian rhythm	14	5	0.69	0.00038	
	GO:0031326 regulation of cellular biosynthetic proc...	586	47	28.68	0.00043	
	GO:0019219 regulation of nucleobase-containing comp...	587	47	28.72	0.00044	
	GO:0051171 regulation of nitrogen compound metaboli...	588	47	28.77	0.00046	
	GO:0080090 regulation of primary metabolic process	655	51	32.05	0.00048	
	GO:0009889 regulation of biosynthetic process	591	47	28.92	0.00052	
	GO:0051252 regulation of RNA metabolic process	576	46	28.19	0.00054	
	GO:2000112 regulation of cellular macromolecule bio...	576	46	28.19	0.00054	
	GO:0048511 rhythmic process	15	5	0.73	0.00054	
	GO:0010556 regulation of macromolecule biosynthetic...	577	46	28.24	0.00056	
	GO:0006355 regulation of transcription, DNA-templat...	562	45	27.5	0.00059	
	GO:1903506 regulation of nucleic acid-templated tra...	562	45	27.5	0.00059	
	GO:2001141 regulation of RNA biosynthetic process	562	45	27.5	0.00059	
	GO:0060255 regulation of macromolecule metabolic pr...	678	52	33.18	0.00061	
	GO:0031323 regulation of cellular metabolic process	664	51	32.49	0.00066	
	GO:0010468 regulation of gene expression	618	48	30.24	0.00076	
	GO:0019222 regulation of metabolic process	732	54	35.82	0.00119	
	GO:0007165 signal transduction	1329	87	65.03	0.00163	
	GO:0032922 circadian regulation of gene expression	6	3	0.29	0.00208	
	GO:0006351 transcription, DNA-templated	639	47	31.27	0.00269	
	GO:0097659 nucleic acid-templated transcription	639	47	31.27	0.00269	
	GO:0032774 RNA biosynthetic process	641	47	31.37	0.00286	
	GO:0044700 single organism signaling	1356	87	66.36	0.00299	
	GO:0007154 cell communication	1379	88	67.48	0.0033	
	GO:0023052 signaling	1363	87	66.7	0.00348	

Cluster V	GO:0060217	hemangioblast cell differentiation	7	3	0.34	0.00351	
	GO:0021536	diencephalon development	33	6	1.61	0.00474	
	GO:0048333	mesodermal cell differentiation	15	4	0.73	0.005	
	GO:0050896	response to stimulus	1897	114	92.83	0.00558	
	GO:0033292	T-tubule organization	3	2	0.15	0.00693	
	GO:0051716	cellular response to stimulus	1531	94	74.92	0.00732	
	GO:0070121	Kupffer's vesicle development	36	6	1.76	0.00736	
	GO:0008283	cell proliferation	48	7	2.35	0.00823	
	GO:0034654	nucleobase-containing compound biosynthe..	733	50	35.87	0.00878	
	GO:0006874	cellular calcium ion homeostasis	18	4	0.88	0.00999	
	GO:0072503	cellular divalent inorganic cation homeo...	18	4	0.88	0.00999	
	GO:0071910	determination of liver left/right asymme...	10	3	0.49	0.01078	
	GO:1901362	organic cyclic compound biosynthetic pro...	797	53	39	0.0114	
	GO:0001707	mesoderm formation	19	4	0.93	0.01217	
	GO:0019438	aromatic compound biosynthetic process	766	51	37.48	0.01275	
	GO:0042752	regulation of circadian rhythm	4	2	0.2	0.01341	
	GO:0007166	cell surface receptor signaling pathway	506	36	24.76	0.01389	
	GO:0044699	single-organism process	4684	248	229.21	0.01405	
	GO:0007224	smoothened signaling pathway	30	5	1.47	0.01414	
	GO:0071907	determination of digestive tract left/ri...	11	3	0.54	0.01429	
	GO:0007009	plasma membrane organization	20	4	0.98	0.01464	
	GO:0055074	calcium ion homeostasis	20	4	0.98	0.01464	
	GO:0044271	cellular nitrogen compound biosynthetic ...	775	51	37.92	0.01572	
	GO:0048732	gland development	122	12	5.97	0.01587	
	GO:0018130	heterocycle biosynthetic process	778	51	38.07	0.01682	
	GO:0048794	swim bladder development	12	3	0.59	0.01837	
	GO:0044707	single-multicellular organism process	1917	111	93.81	0.0201	
	GO:0006979	response to oxidative stress	22	4	1.08	0.02047	
	GO:0060215	primitive hemopoiesis	33	5	1.61	0.02091	
	GO:0001778	plasma membrane repair	5	2	0.24	0.02164	
	GO:0010955	negative regulation of protein processin...	5	2	0.24	0.02164	
	GO:0021761	limbic system development	5	2	0.24	0.02164	
	GO:0021854	hypothalamus development	5	2	0.24	0.02164	
	GO:0030865	cortical cytoskeleton organization	5	2	0.24	0.02164	
	GO:0030866	cortical actin cytoskeleton organization	5	2	0.24	0.02164	
	GO:0048796	swim bladder maturation	5	2	0.24	0.02164	
	GO:0048798	swim bladder inflation	5	2	0.24	0.02164	
	GO:0048799	organ maturation	5	2	0.24	0.02164	
	GO:0070613	regulation of protein processing	5	2	0.24	0.02164	
	GO:0072507	divalent inorganic cation homeostasis	23	4	1.13	0.02385	
	GO:0032501	multicellular organismal process	1933	111	94.59	0.02543	
	GO:0035270	endocrine system development	47	6	2.3	0.02593	
	GO:0001704	formation of primary germ layer	24	4	1.17	0.02755	
	GO:0048332	mesoderm morphogenesis	24	4	1.17	0.02755	
	GO:0003206	cardiac chamber morphogenesis	14	3	0.69	0.02828	
	GO:0030223	neutrophil differentiation	14	3	0.69	0.02828	
	GO:0070588	calcium ion transmembrane transport	14	3	0.69	0.02828	
	GO:0048856	anatomical structure development	1904	109	93.17	0.02933	
	GO:0009628	response to abiotic stimulus	104	10	5.09	0.03037	
	GO:0003208	cardiac ventricle morphogenesis	6	2	0.29	0.03142	
	GO:0003314	heart rudiment morphogenesis	6	2	0.29	0.03142	
	GO:0003315	heart rudiment formation	6	2	0.29	0.03142	
	GO:0006000	fructose metabolic process	6	2	0.29	0.03142	
	GO:0035108	limb morphogenesis	6	2	0.29	0.03142	
	GO:0045880	positive regulation of smoothened signal...	6	2	0.29	0.03142	
	GO:0050935	iridophore differentiation	6	2	0.29	0.03142	
	GO:0006875	cellular metal ion homeostasis	25	4	1.22	0.03157	
	GO:0006873	cellular ion homeostasis	26	4	1.27	0.03592	
	GO:0030003	cellular cation homeostasis	26	4	1.27	0.03592	
	GO:0055082	cellular chemical homeostasis	26	4	1.27	0.03592	
	GO:0007275	multicellular organismal development	1806	103	88.38	0.03817	
	GO:0001964	startle response	7	2	0.34	0.04258	
	GO:0003231	cardiac ventricle development	7	2	0.34	0.04258	
	GO:0008285	negative regulation of cell proliferatio...	7	2	0.34	0.04258	
	GO:0060173	limb development	7	2	0.34	0.04258	
	GO:0042692	muscle cell differentiation	126	11	6.17	0.04394	
	GO:0055123	digestive system development	82	8	4.01	0.0464	
	GO:0007498	mesoderm development	54	6	2.64	0.04704	
	GO:0030900	forebrain development	68	7	3.33	0.04744	
	GO:0032502	developmental process	1961	110	95.96	0.04851	
Cluster VI	GO:0044710	single-organism metabolic process	638	24	12.7	0.00092	49
	GO:0006869	lipid transport	25	4	0.5	0.00133	
	GO:0010876	lipid localization	26	4	0.52	0.00155	
	GO:0072378	blood coagulation, fibrin clot formation	4	2	0.08	0.00229	
	GO:0042060	wound healing	53	5	1.05	0.00372	
	GO:0055114	oxidation-reduction process	258	12	5.14	0.0044	
	GO:0002066	columnar/cuboidal epithelial cell develo...	6	2	0.12	0.00557	
	GO:0009072	aromatic amino acid family metabolic pro...	6	2	0.12	0.00557	
	GO:0072376	protein activation cascade	6	2	0.12	0.00557	
	GO:0009611	response to wounding	62	5	1.23	0.00731	
	GO:0008610	lipid biosynthetic process	41	4	0.82	0.0084	
	GO:0031018	endocrine pancreas development	12	2	0.24	0.02268	
	GO:0010639	negative regulation of organelle organiz...	13	2	0.26	0.02646	
	GO:0043207	response to external biotic stimulus	35	3	0.7	0.03143	
	GO:0051707	response to other organism	35	3	0.7	0.03143	
	GO:0000280	nuclear division	15	2	0.3	0.03472	
	GO:0009607	response to biotic stimulus	37	3	0.74	0.03627	
	GO:0071383	cellular response to steroid hormone sti...	38	3	0.76	0.03883	
	GO:0043414	macromolecule methylation	16	2	0.32	0.03918	
	GO:0000281	mitotic cytokinesis	2	1	0.04	0.03942	
	GO:0002067	glandular epithelial cell differentiatio...	2	1	0.04	0.03942	
	GO:0002068	glandular epithelial cell development	2	1	0.04	0.03942	
	GO:0006568	tryptophan metabolic process	2	1	0.04	0.03942	
	GO:0006569	tryptophan catabolic process	2	1	0.04	0.03942	
	GO:0006576	cellular biogenic amine metabolic proces...	2	1	0.04	0.03942	
	GO:0006586	indolalkylamine metabolic process	2	1	0.04	0.03942	
	GO:0009074	aromatic amino acid family catabolic pro...	2	1	0.04	0.03942	
	GO:0009308	amine metabolic process	2	1	0.04	0.03942	
	GO:0009310	amine catabolic process	2	1	0.04	0.03942	
	GO:0010960	magnesium ion homeostasis	2	1	0.04	0.03942	
	GO:0030212	hyaluronan metabolic process	2	1	0.04	0.03942	
	GO:0035883	enteroendocrine cell differentiation	2	1	0.04	0.03942	
	GO:0042402	cellular biogenic amine catabolic proces...	2	1	0.04	0.03942	
	GO:0042436	indole-containing compound catabolic pro...	2	1	0.04	0.03942	
	GO:0042446	hormone biosynthetic process	2	1	0.04	0.03942	
	GO:0044106	cellular amine metabolic process	2	1	0.04	0.03942	
	GO:0046218	indolalkylamine catabolic process	2	1	0.04	0.03942	
	GO:0050910	detection of mechanical stimulus involve...	2	1	0.04	0.03942	
	GO:0050974	detection of mechanical stimulus involve...	2	1	0.04	0.03942	
	GO:0050982	detection of mechanical stimulus	2	1	0.04	0.03942	
	GO:0061640	cytoskeleton-dependent cytokinesis	2	1	0.04	0.03942	
	GO:0070059	intrinsic apoptotic signaling pathway in...	2	1	0.04	0.03942	
	GO:1903510	mucopolysaccharide metabolic process	2	1	0.04	0.03942	
	GO:0065008	regulation of biological quality	167	7	3.32	0.04675	
	GO:0032870	cellular response to hormone stimulus	41	3	0.82	0.04705	
	GO:0048545	response to steroid hormone	41	3	0.82	0.04705	
	GO:0071396	cellular response to lipid	41	3	0.82	0.04705	
	GO:0048285	organelle fission	18	2	0.36	0.0487	
	GO:0071407	cellular response to organic cyclic comp...	42	3	0.84	0.04997	
