# Supplementary material for: Transgenerational effects persist down the maternal line in marine sticklebacks: gene expression matches physiology in a warming ocean
Source: Evol Appl. 2016 Feb 28;9(9):1096–111. doi: 10.1111/eva.12370 (PMC5039323; doi:10.1111/eva.12370)
Supplement: Supplementary file 4 — Table S4. Genes representing enriched processes with their respective ensembl gene ID, name and description. [file EVA-9-1096-s004.pdf]

Genes representing enriched processes

ensembl_gene_id	external_gene_name	description
ENSGACG00000015514	LGR4	leucine-rich repeat containing G protein-coupled receptor 4 [Source:HGNC Symbol;Acc:HGNC:13299]
ENSGACG00000007741	gnai3	guanine nucleotide binding protein (G protein), alpha inhibiting activity polypeptide 3 [Source:ZFIN;Acc:ZDB-GENE-070713-6]
ENSGACG00000003291	gpr161	G protein-coupled receptor 161 [Source:ZFIN;Acc:ZDB-GENE-030616-58]
ENSGACG00000011440	gprc5ba	G protein-coupled receptor, class C, group 5, member Ba [Source:ZFIN;Acc:ZDB-GENE-030131-8162]
ENSGACG00000016116	rgs13	regulator of G-protein signaling 13 [Source:ZFIN;Acc:ZDB-GENE-070410-138]
ENSGACG00000009446	slc1a5	solute carrier family 1 (neutral amino acid transporter), member 5 [Source:ZFIN;Acc:ZDB-GENE-070501-4]
ENSGACG00000011861	SLC7A1 (1 of 2)	solute carrier family 7 (cationic amino acid transporter, y+ system), member 1 [Source:HGNC Symbol;Acc:HGNC:11057]
ENSGACG00000019161	slc7a8a	solute carrier family 7 (amino acid transporter light chain, L system), member 8a [Source:ZFIN;Acc:ZDB-GENE-121120-2]
ENSGACG00000017222	slc20a1b	solute carrier family 20 (phosphate transporter), member 1b [Source:ZFIN;Acc:ZDB-GENE-030131-260]
ENSGACG00000020037	cry1aa	cryptochrome circadian clock 1aa [Source:ZFIN;Acc:ZDB-GENE-010426-2]
ENSGACG00000019308	per1b	period circadian clock 1b [Source:ZFIN;Acc:ZDB-GENE-040419-1
ENSGACG00000005754	mrpl32	mitochondrial ribosomal protein L32 [Source:ZFIN;Acc:ZDB-GENE-060825-188]
ENSGACG00000020492	mrps17	mitochondrial ribosomal protein S17 [Source:ZFIN;Acc:ZDB-GENE-050522-390]
ENSGACG00000009992	mrpl47	mitochondrial ribosomal protein L47 [Source:ZFIN;Acc:ZDB-GENE-030219-177]
ENSGACG00000010541	mrpl12	mitochondrial ribosomal protein L12 [Source:ZFIN;Acc:ZDB-GENE-050417-187]
ENSGACG00000012264	mrps7	mitochondrial ribosomal protein S7 [Source:ZFIN;Acc:ZDB-GENE-050809-112]
ENSGACG00000006884	tfb1m	transcription factor B1, mitochondrial [Source:ZFIN;Acc:ZDB-GENE-060929-1010]
ENSGACG00000001488	UTP14A	UTP14, U3 small nucleolar ribonucleoprotein, homolog A (yeast) [Source:HGNC Symbol;Acc:HGNC:10665]
ENSGACG00000019392	YBX2	Y box binding protein 2 [Source:HGNC Symbol;Acc:HGNC:17948]
ENSGACG00000016147	tbp	TATA box binding protein [Source:ZFIN;Acc:ZDB-GENE-030616-563]
ENSGACG00000000309	gnl3	guanine nucleotide binding protein-like 3 (nucleolar) [Source:ZFIN;Acc:ZDB-GENE-030131-616]
ENSGACG00000011997	atf3	activating transcription factor 3 [Source:ZFIN;Acc:ZDB-GENE-040426-728]
ENSGACG00000003829	tbx21	T-box 21 [Source:ZFIN;Acc:ZDB-GENE-080104-3]
ENSGACG00000006880	gtf2f2a	general transcription factor IIF, polypeptide 2a [Source:ZFIN;Acc:ZDB-GENE-040625-131]
ENSGACG00000005341	spen	spen family transcriptional repressor [Source:ZFIN;Acc:ZDB-GENE-050309-70]
ENSGACG00000000451	nr4a1	nuclear receptor subfamily 4, group A, member 1 [Source:ZFIN;Acc:ZDB-GENE-040704-11]
ENSGACG00000009027	nr4a3	nuclear receptor subfamily 4, group A, member 3 [Source:ZFIN;Acc:ZDB-GENE-070824-4]
ENSGACG00000008896	nr5a2	nuclear receptor subfamily 5, group A, member 2 [Source:ZFIN;Acc:ZDB-GENE-990415-79]
ENSGACG00000010803	acot8	acyl-CoA thioesterase 8 [Source:ZFIN;Acc:ZDB-GENE-041010-174]
ENSGACG00000008374	mat2ab	methionine adenosyltransferase II, alpha b [Source:ZFIN;Acc:ZDB-GENE-050327-6]
ENSGACG00000019043	pycr1b	pyrroline-5-carboxylate reductase 1b [Source:ZFIN;Acc:ZDB-GENE-050522-26]
ENSGACG00000015018	hdc	histidine decarboxylase [Source:ZFIN;Acc:ZDB-GENE-080102-5]
ENSGACG00000010678	acsf2 (2 of 2)	acyl-CoA synthetase family member 2 [Source:ZFIN;Acc:ZDB-GENE-060825-7]
ENSGACG00000015439	ACSF3	acyl-CoA synthetase family member 3 [Source:HGNC Symbol;Acc:HGNC:27288]
ENSGACG00000020877	ACSL6	acyl-CoA synthetase long-chain family member 6 [Source:HGNC Symbol;Acc:HGNC:16496]
ENSGACG00000009167	idh1	isocitrate dehydrogenase 1 (NADP+), soluble [Source:ZFIN;Acc:ZDB-GENE-031006-1]
ENSGACG00000008812	ACLY (2 of 2)	ATP citrate lyase [Source:HGNC Symbol;Acc:HGNC:115]
ENSGACG00000007279	me2	malic enzyme 2, NAD(+)-dependent, mitochondrial [Source:ZFIN;Acc:ZDB-GENE-040801-147]
ENSGACG00000007196	creb5a	cAMP responsive element binding protein 5a [Source:ZFIN;Acc:ZDB-GENE-120827-2]
ENSGACG00000002417	tomm40l	translocase of outer mitochondrial membrane 40 homolog, like [Source:ZFIN;Acc:ZDB-GENE-040426-2319]
ENSGACG00000010817	tdh	L-threonine dehydrogenase [Source:ZFIN;Acc:ZDB-GENE-040426-2379]
ENSGACG00000005410	cyp46a1.2	cytochrome P450, family 46, subfamily A, polypeptide 1, tandem duplicate 2 [Source:ZFIN;Acc:ZDB-GENE-040426-1184]
ENSGACG00000017590	crema	cAMP responsive element modulator a [Source:ZFIN;Acc:ZDB-GENE-030131-7031]
ENSGACG00000011270	ldha	lactate dehydrogenase A4 [Source:ZFIN;Acc:ZDB-GENE-991026-5]
ENSGACG00000017414	cyp8b1	cytochrome P450, family 8, subfamily B, polypeptide 1 [Source:ZFIN;Acc:ZDB-GENE-040808-53]
ENSGACG00000012860	creb3l3a	cAMP responsive element binding protein 3-like 3a [Source:ZFIN;Acc:ZDB-GENE-030131-4298]
ENSGACG00000002457	cyb5r2	cytochrome b5 reductase 2 [Source:ZFIN;Acc:ZDB-GENE-060825-83]
ENSGACG00000016669	cyp3a65 (1 of 3)	cytochrome P450, family 3, subfamily A, polypeptide 65 [Source:ZFIN;Acc:ZDB-GENE-050604-1]
ENSGACG00000016600	pkma	pyruvate kinase, muscle, a [Source:ZFIN;Acc:ZDB-GENE-031201-4]
ENSGACG00000014674	cyp1a	cytochrome P450, family 1, subfamily A [Source:ZFIN;Acc:ZDB-GENE-011219-1]
ENSGACG00000008226	rars2	arginyl-tRNA synthetase 2, mitochondrial (putative) [Source:ZFIN;Acc:ZDB-GENE-040426-1244]
ENSGACG00000006474	mars	methionyl-tRNA synthetase [Source:ZFIN;Acc:ZDB-GENE-030219-83]
ENSGACG00000007438	mto1	mitochondrial tRNA translation optimization 1 [Source:ZFIN;Acc:ZDB-GENE-070209-253]
ENSGACG00000000697	tsfm	Ts translation elongation factor, mitochondrial [Source:ZFIN;Acc:ZDB-GENE-061215-17]
ENSGACG00000007099	lars2	leucyl-tRNA synthetase 2, mitochondrial [Source:ZFIN;Acc:ZDB-GENE-070928-3]
ENSGACG00000006060	trmt11	tRNA methyltransferase 11 homolog (S. cerevisiae) [Source:ZFIN;Acc:ZDB-GENE-040426-953]
ENSGACG00000003720	yars2	tyrosyl-tRNA synthetase 2, mitochondrial [Source:ZFIN;Acc:ZDB-GENE-030131-6268]
ENSGACG00000014220	mtfmt	mitochondrial methionyl-tRNA formyltransferase [Source:ZFIN;Acc:ZDB-GENE-060929-120]
ENSGACG00000005820	sars2	seryl-tRNA synthetase 2, mitochondrial [Source:ZFIN;Acc:ZDB-GENE-040426-1616]
ENSGACG00000012875	hsp90aa1.1	heat shock protein 90, alpha (cytosolic), class A member 1, tandem duplicate 1 [Source:ZFIN;Acc:ZDB-GENE-990415-94]
ENSGACG00000008995	hspe1	heat shock 10 protein 1 [Source:ZFIN;Acc:ZDB-GENE-000906-2]
ENSGACG00000015472	f7i	coagulation factor VIIi [Source:ZFIN;Acc:ZDB-GENE-021206-10]
ENSGACG00000018674	fgb	fibrinogen beta chain [Source:ZFIN;Acc:ZDB-GENE-030131-9261]
ENSGACG00000018694	fga	fibrinogen alpha chain [Source:ZFIN;Acc:ZDB-GENE-031010-21]
ENSGACG00000020449	f9b	coagulation factor IXb [Source:ZFIN;Acc:ZDB-GENE-060421-7346]
ENSGACG00000015767	proca	protein C (inactivator of coagulation factors Va and VIIIa), a [Source:ZFIN;Acc:ZDB-GENE-060824-5]
ENSGACG00000010566	perp	PERP, TP53 apoptosis effector [Source:ZFIN;Acc:ZDB-GENE-050104-1]
ENSGACG00000007426	CARD10	caspase recruitment domain family, member 10 [Source:HGNC Symbol;Acc:HGNC:16422]
ENSGACG00000015843	tnfrsfa	tumor necrosis factor receptor superfamily, member a [Source:ZFIN;Acc:ZDB-GENE-010802-1]
ENSGACG00000006480	ddit3	DNA-damage-inducible transcript 3 [Source:ZFIN;Acc:ZDB-GENE-070410-90]
ENSGACG00000011826	NPC1L1	NPC1-like 1 [Source:HGNC Symbol;Acc:HGNC:7898]
ENSGACG00000012469	CABIN1	calcineurin binding protein 1 [Source:HGNC Symbol;Acc:HGNC:24187]
ENSGACG00000009700	CHST15 (2 of 2)	carbohydrate (N-acetylgalactosamine 4-sulfate 6-O) sulfotransferase 15 [Source:HGNC Symbol;Acc:HGNC:18137]
ENSGACG00000001955	cdc16	cell division cycle 16 homolog (S. cerevisiae) [Source:ZFIN;Acc:ZDB-GENE-051113-132]
